# A Sacrificial Linker in Biodegradable Polyesters for Accelerated Photoinduced Degradation, Monitored by Continuous Atline SEC Analysis

**DOI:** 10.1021/acsmacrolett.4c00117

**Published:** 2024-04-16

**Authors:** Samuel B. H. Patterson, Valeria Arrighi, Filipe Vilela

**Affiliations:** A Samuel B. H. Patterson - School of Engineering and Physical Sciences, Institute of Chemical Sciences, Heriot Watt University, Edinburgh EH14 4AS, U.K.; B Valeria Arrighi - School of Engineering and Physical Sciences, Institute of Chemical Sciences, Heriot Watt University, Edinburgh EH14 4AS, U.K.; C Filipe Vilela - School of Engineering and Physical Sciences, Institute of Chemical Sciences, Heriot Watt University, Edinburgh EH14 4AS, U.K.

## Abstract

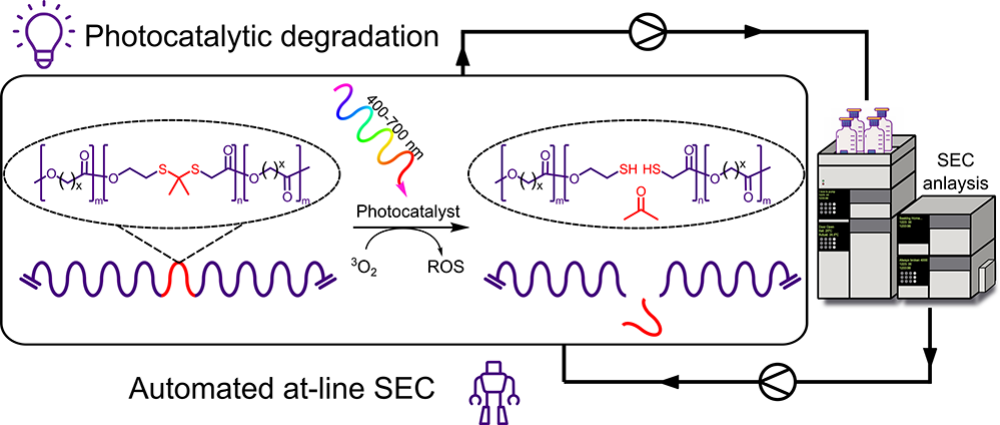

Polymeric materials that undergo photoinduced degradation have wide application in fields such as controlled release. Most methods for photoinduced degradation rely on the UV or near-UV region of the electromagnetic spectrum; however, use of the deeply penetrating and benign wavelengths of visible light offers a multitude of advantages. Here we report a lactone monomer for ring-opening copolymerizations to introduce a sacrificial linker into a polymer backbone which can be cleaved by reactive oxygen species which are produced by a photocatalyst under visible light irradiation. We find that copolymers of this material readily degrade under visible light. We followed polymer degradation using a continuous flow size exclusion chromatography system, the components of which are described herein.

The accelerated degradation of polymeric materials is a promising area of research which has grown in the past few decades as a result of the demand for greener and “benign by design” materials.^1,2^ Polymers such as poly(lactic acid),^3,4^ poly(ε-caprolactone),^5,6^ and naturally derived biopolymers such as cellulose^7−12^ and chitin^13−17^ are often at the forefront of materials used in this research. However, the mode of degradation of these polymers relies on variable factors such as enzymatic activity^18,19^ or pH.^20−22^ Where degradation is desirable, such pathways can limit the utility of these polymers in areas where these stimuli are unavailable or in cases where the degradation rate afforded by the stimuli mentioned above does not occur on a sufficient time scale.

To take the case of biomedical research, accelerated photoinduced degradation (APD) has received considerable attention for drug delivery.^23−26^ APD relies on molecules that undergo changes to their physiochemical structure when irradiated by light of a specific wavelength. Research in this area is largely focused on irradiation by ultraviolet light;^27−29^ however, in many cases the use of visible light can offer a multitude of advantages over UV light. For example, in situations where APD is performed in living tissue, the deeply penetrating and benign wavelengths of visible light are clearly beneficial.^30^

The availability of materials which undergo APD in the visible region are not as common as those active in UV.^31^ This follows when one considers the high-energy photons needed for the cleavage of covalent bonds. A method to circumvent this high energy requirement is to use an indirect approach of photostimulation. An example of this is the use of functional groups in a polymer chain which show selective reactivity toward reactive oxygen species (ROS), resulting in bond cleavage.^32^ ROS can be produced by a photocatalyst, active in the visible region.^33^ The dependence of the wavelength of absorption thus shifts from the polymer material to the maximum absorption wavelength (λ_max_) of the photocatalyst used.

In our previous research we have shown how electron donor–acceptor systems based on the benzo[c][1,2,5]thiadiazole (BTZ) core can be systematically modified to alter the photocatalyst’s optoelectronic and photophysical properties, producing a library of photocatalysts with λ_max_ ranging from 359–505 nm.^34−41^

In this research, our goal was to generate an analogue of the cyclic esters which are the common feedstock for biodegradable polymers, typically formed by ring-opening polymerizations (ROPs). Incorporation of a selectively oxidized functional group (SOFG) into the analogue produces a cyclic ester which can be copolymerized with other ring opening monomers (Figure 1a). This SOFG provides a sacrificial linker at random points along a polymer chain, allowing the scission of the chain and promoting accelerated degradation (Figure 1b). Our choice of SOFG was inspired by the work of Cao et al. with their ROS-responsive nanomaterials for red or near-infrared light activated drug release.^42^ The thioketal (TK) group employed is readily cleaved by ROS in the presence of (i) a source of molecular oxygen, (ii) a photocatalyst, and (iii) a light source of a suitable wavelength.^43−45^ The mechanism for the cleavage of the thioketal functional group has been shown to proceed via superoxide, peroxide, and singlet oxygen ROS.^42,45,46^ Literature reports in the use of the TK group have also shown that the thiol and acetone degradation products of oxidation are nontoxic.^43^ Here, we report on the synthesis of a TK-containing monomer, 5,5-dimethyl-1,4,6-oxadithiocan-2-one (DMODT), for incorporation into a biodegradable copolymer (Figure 1c).

**Figure 1:**
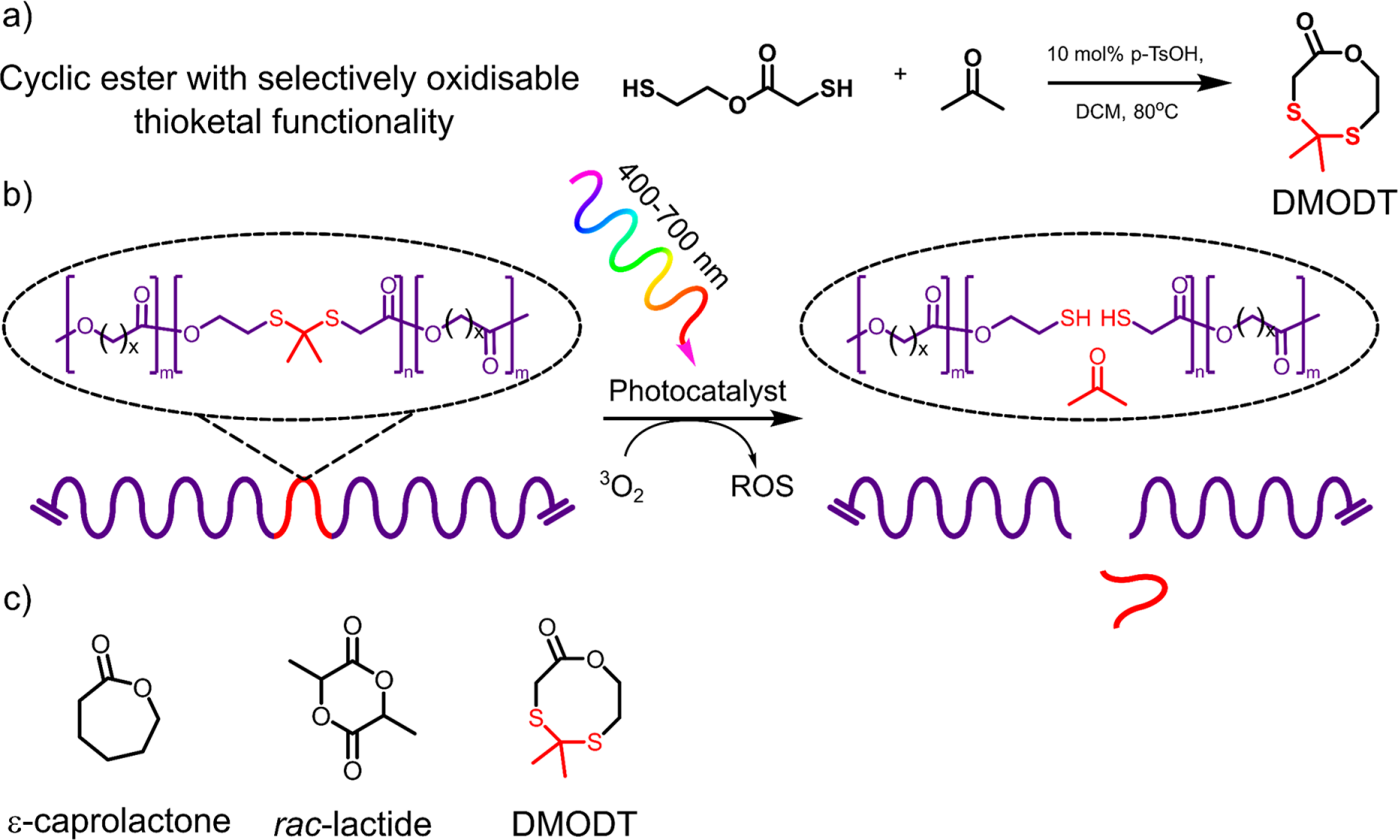
(a) Synthesis of monomer DMODT containing selectively oxidizable thioketal functionality, (b) polymer chain showing the degradation products (thiol groups and acetone) after visible light irradiation in the presence of oxygen and a photocatalyst, (c) scope for copolymerization with other cyclic esters.

Herein we report on the successful copolymerization of DMODT with ε-caprolactone. Size exclusion chromatography (SEC, also referred to as gel permeation chromatography, GPC) was used to follow the progress of polymer degradation as scission of the copolymer backbone resulted in a change in distribution of average polymer molecular weight. This showed that the resultant materials were readily oxidized by ROS.

In previous work, we have shown a simple method of integrating SEC with a commercially available flow reactor to monitor the enzymatic degradation of poly(ε-caprolactone).^47^ This system of atline analysis provides an automated, repeatable, and time efficient means to monitor accelerated polymer degradation over time. Coupled with this, the utility of continuous flow when applied to photochemistry enables the most effective reaction conditions and ease of controlling variables.^48^

Full synthetic procedures for the synthesis of DMODT can be found in section 2 of the Supporting Information. It should be noted that synthesis of a 7-membered analogue of ε-caprolactone containing disulfide functionality (1,4,5-oxadithiepan-2-one (OTP)) was performed before DMODT was synthesized. This species had previously been reported in literature and is shown in section 2.2 of the Supporting Information.^49^ However, OTP proved to be unstable toward ROP but provided inspiration for the synthesis of DMODT. Initially, we were able to show the successful oxidation of DMODT monomer by ^1^H NMR over 24 h with 420 nm irradiation using 4,7-diphenylbenzo[c][1,2,5]thiadiazole (DiPh-BTZ) as photocatalyst at 5 mol % loading. Oxidation of the TK group produces acetone which shows as a singlet in the ^1^H NMR at 2.17 ppm (CDCl_3_ solvent).^50^ Following the formation of this signal over 24 h and comparing the signal integration with that of the 2 methyl groups of DMODT gave a final conversion of 13.3%. At this point, the reaction is limited by the availability of molecular oxygen and the photobleaching of the photocatalyst. The same experiment was performed with negative control samples, ultimately showing that the oxidation proceeded only in the presence of light, oxygen, and photocatalyst.

Following the synthesis of DMODT, we selected two typical precursors for biodegradable polymers to copolymerize with ε-caprolactone and *rac-*lactide. The range of mol % loading of initial DMODT monomer copolymerized was 5, 10, 25, and 50 mol % for ε-caprolactone and 25 and 50 mol % for *rac*-lactide. Copolymerization with *rac-*lactide was attempted to demonstrate the versatility of DMODT as a ring-opening comonomer; however, under the experimental conditions used, copolymerization with *rac-*lactide only produced low molecular weight oligomers. All subsequent analysis is focused on copolymers of ε-caprolactone, unless otherwise stated. We reasoned that the inclusion of a higher mol % loading of initial DMODT monomer for random copolymerizations would lead to an increase in the number of scission points along the copolymer chain and therefore a greater change in molecular weight after oxidation. The actual amount of DMODT contained in the copolymer chains was established by integration of the ^1^H NMR signals (CDCl_3_ solvent) between δ_ppm_ = 2.88 (t, 2H, OCH_2_C**H**_**2**_S) of the poly(DMODT) segments and δ_ppm_ = 2.31 (t, 2H, CH_2_C**H**_**2**_C=O) of the poly(ε-caprolactone) segments. The copolymer composition for each copolymerization with ε-caprolactone performed in triplicate is tabulated in Figure 2b.

**Figure 2:**
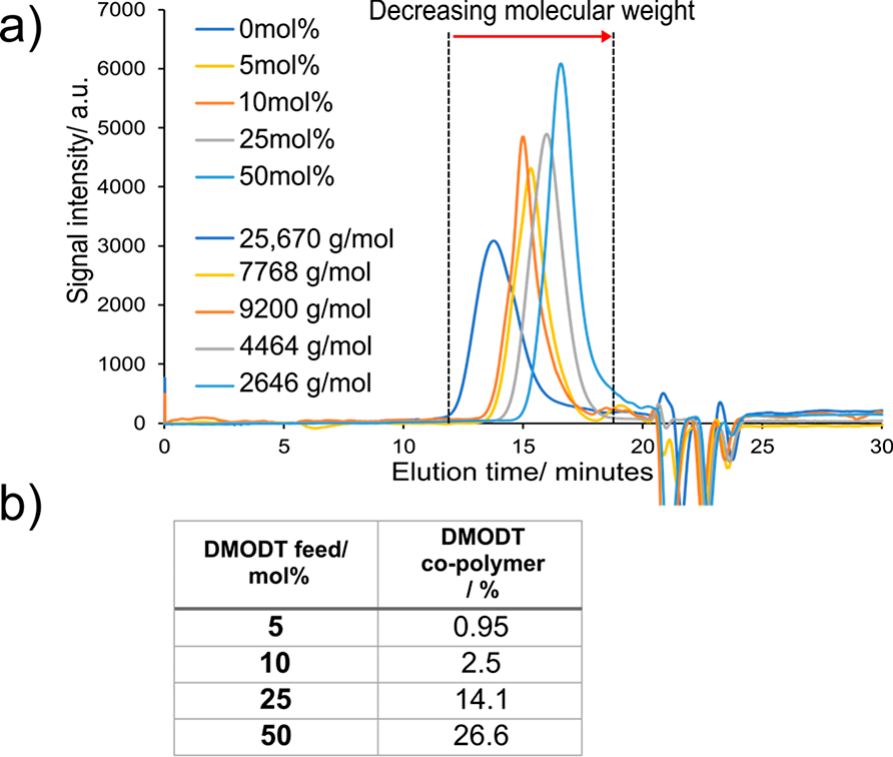
(a) SEC chromatograms of poly(DMODT-*co*-caprolactone) showing a decrease in number-average molecular weight proportional to the initial mol % of DMODT (0–50 mol %) monomer used in the polymerizations. (b) The feed composition vs the copolymer composition of DMODT into copolymers of poly(DMODT-*co*-caprolactone) is shown in the table, calculated by comparison of ^1^H NMR peak integrations between δ_ppm_ = 2.88 (t, 2H, OCH_2_C**H**_**2**_S) of poly(DMODT) segments and δ_ppm_ = 2.31 (t, 2H, CH_2_C**H**_**2**_C=O) of polycaprolactone segments.

The difference in DMODT content in the copolymer as compared to the initial feed composition of the polymerization reaction could be a result of the different reactivity ratios between the two monomers.^51^ Another explanation is DMODT degrades at elevated temperatures such as those required for the Sc(Oct)2 ROP catalyst employed.^52^ This would likely have an influence on the final polymer copolymer composition. After copolymerizations with ε-caprolactone, the filtrate from polymer precipitation was evaporated to dryness and the resultant residue was analyzed by ^1^H NMR. The spectra did not show any remaining DMODT monomer but did show residual ε-caprolactone, supporting our claim that DMODT which was not included in the copolymer underwent thermal degradation. Thermal gravimetric analysis showed an endothermic peak corresponding to the thermal degradation of DMODT beginning at around 130 °C (Figure S13 of the Supporting Information). Copolymerizations with a mol % loading of DMODT greater than 50 mol % failed to polymerize and were discounted from further experiments. Following the loss of DMODT monomer during standard copolymerization conditions described in section 3 of the Supporting Information a marked difference in molecular weight of the resulting copolymers was observed. Figure 2a shows the SEC traces of copolymers of poly(DMODT)-*co*-caprolactone, each with differing mol % loading of initial DMODT monomer.

The proportional decrease in molecular weight with increasing DMODT points to the thermal degradation of the DMODT monomer outlined above. An alternate catalyst system which requires milder reaction conditions may result in a higher molecular weight for the copolymers.^52^ This line of research will be pursued in future work.

To prove the successful incorporation of DMODT into the copolymer chain, as opposed to DMODT monomer being physically entrapped within the matrix of a homopolymer of poly(ε-caprolactone), diffusion-ordered spectroscopy (DOSY) NMR was used. The diffusion coefficients calculated from the ^1^H NMR signals of each copolymer were within close agreement to each other. This confirms that the polymerized DMODT and the corresponding copolymer diffuse at the same rate and are therefore part of the same copolymer chain (Section 5.2.2 of the Supporting Information). Copolymer samples containing DMODT were kept under standard conditions for 2 weeks in a sealed vial and showed no sign of degradation by ^1^H NMR.

The degradation profile of the copolymers was analyzed in a continuous mode via atline SEC, at sampling resolution of two samples per hour over an extended period of time. The flow reaction setup was easily modified from that used in our previous work,^47^ highlighting the versatility of this system. In brief, a 0.5 wt % solution of the copolymer and 0.1 wt % photocatalyst loading (with respect to the copolymer) was prepared in HPLC grade THF. This solution was pumped at 1 mL min^–1^ through a 20 μL sample coil on a two-position automated valve before being mixed with a slug flow of air (also pumped at 1 mL min^–1^), presaturated with THF to minimize solvent evaporation.

The two-phase solution then flowed through a 10 mL photoreactor coil irradiated by 420 nm light before being returned to the bulk solution. At 30 min intervals, the automated valve switched positions to allow SEC eluent to wash the contents of the sample coil on the SEC column for analysis. This cycle was repeated over 24 h (Figure 3). The SEC analysis showed that the early elution peak corresponding to the copolymer reduced in area over 24 h, with gradual appearance of oligomer peaks at long elution times. Figure S14 of the Supporting Information shows the initial and final SEC traces after 24 h.

**Figure 3:**
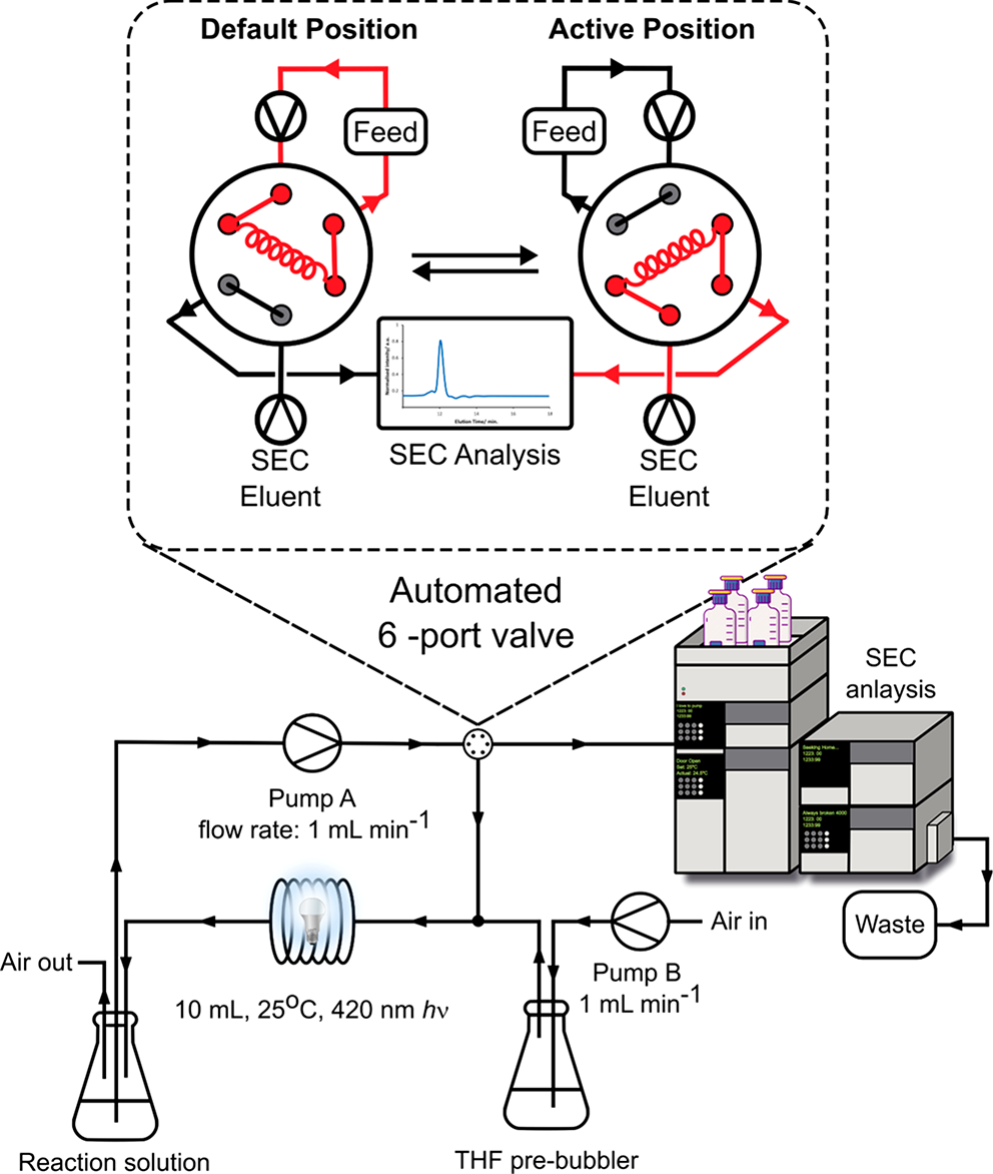
Continuous atline SEC monitoring system for photocatalytic induced oxidation of poly(DMODT)-*co*-caprolactone with detail of the 6-port, 2-position valve indicating how the reaction solution (feed) is sampled at 30 min intervals over a 24-h period.

The continuous flow analysis system (Figure 3) ensured that the concentration of the copolymer-photocatalyst solution remained constant over 24 h. Only the total volume of the solution decreased as a 20 μL aliquot was taken every 30 min (0.96 mL over 24 h). Therefore, any change in peak area for early elution peaks is a direct result of a decrease in the amount of copolymer left in solution. This is reinforced by the late elution peak area being proportional to the area lost by the early elution peak. The position of the early elution peak remained relatively constant with all samples, indicating that after 24 h there remains enough of the initial copolymer to produce a signal in the SEC trace. The reduction in the early elution peak area over 24 h was used as a means to compare the degradation profile of each copolymer sample, with the results of the experiments in triplicate shown in Figure 4c.

**Figure 4:**
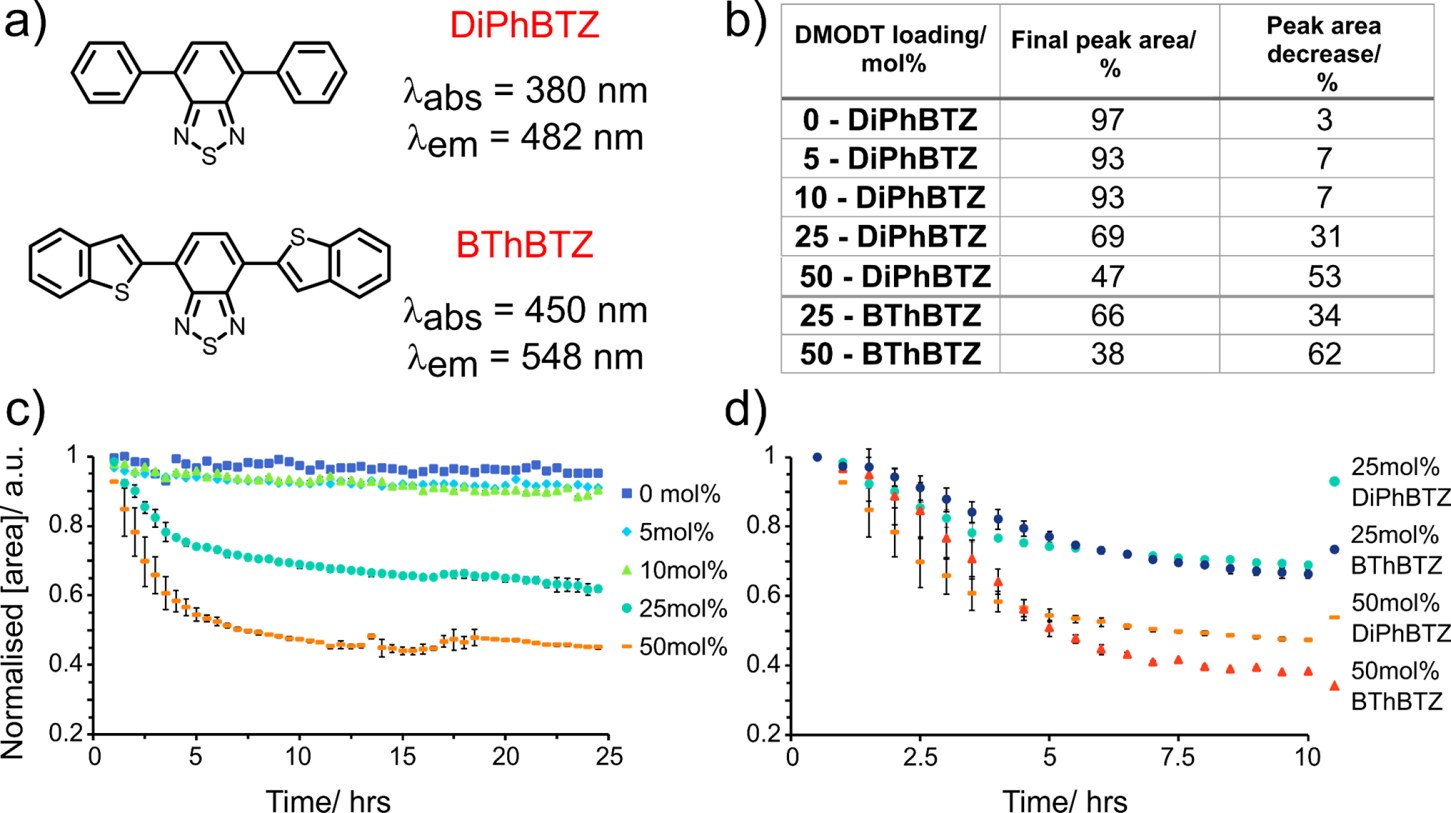
(a) 4,7-Diphenylbenzo[c][1,2,5]thiadiazole (DiPhBTZ) and 4,7-bis(benzo[b]thiophen-2-yl)benzo[c][1,2,5]thiadiazole (BThBTZ) photocatalysts used in this work and maximum absorption and emission wavelengths, (b) comparison of copolymer peak decrease over 10 h as a function of varying DMODT content and a change in photocatalyst, (c) normalized peak area from SEC copolymer elution peak for the different copolymers vs time as a function of varying DMODT content, over 24 h using DiPhBTZ. (d) Normalized peak area decrease of copolymer over 10 h showing a comparison between DiPhBTZ and BThBTZ photocatalysts, BThBTZ leading to a greater decrease in peak area.

The peak area for the control sample of pure poly(ε-caprolactone) remained constant within the standard deviation of the repeat experiments. All other samples containing DMODT showed some level of decrease in the early elution peak area. The 5 and 10 mol % DMODT samples did not show a significant deviation from each other in terms of peak area decrease but did differ significantly from the poly(ε-caprolactone) control. The most significant decrease in early elution peak area can be seen with the 25 and 50 mol % samples. Both samples displayed similar behavior with a rapid decrease in peak area in the first ∼5 h of irradiation before plateauing over the subsequent 19 h. 50 mol % DMODT displayed a greater decrease in peak area in the first 10 h compared to 25 mol % DMODT at 53% decrease and 31% decrease, respectively (Figure 4b). The final peak areas for both samples after 24 h decreased to 38% and 65% for 25 mol % and 50 mol % DMODT, respectively. These results support our initial hypothesis that an increase in DMODT loading in the copolymer chain would result in an increased rate of polymer degradation. The plateauing of peak area decrease observed over 24 h could be explained by two factors. First, the DMODT segments of the polymer chain have been completely oxidized, and second, the photocatalyst may have become photobleached and therefore decreased in potency over time. It was noted in subsequent ^1^H NMR analysis of the samples postirradiation that the DiPh-BTZ photocatalyst used in these experiments was no longer present. The photostability of the photocatalyst has been tested in previous work, and 24 h of irradiation had no affect.^34^ However, irradiation of the sample solutions in continuous flow is a much more efficient process due to the decreased path length of light through 1 mm diameter flow tubing compared to a larger diameter glass NMR tube used in previous photostability experiments. The LED power in the commercial flow reactor is also greater than the lab-made LED modules used previously (60 W compared to 4 × 3 W, respectively). ^1^H NMR analysis of the degradation products can be found in Figure S9 of the Supporting Information, highlighting the potential degradation products including acetone.

To explore the effect of increased irradiation efficiency of the photocatalyst during the photocatalytic oxidation experiment, another photocatalyst 4,7-bis(benzo[b]thiophen-2-yl)benzo[c][1,2,5]thiadiazole (BTh-BTZ) was employed. The absorption peak of BTh-BTZ has a greater overlap with the 420 nm irradiation of the LED lamps used compared with that of DiPh-BTZ (see Figure S1 of the Supporting Information). The photocatalyst loading remained consistent with previous experiments, but only copolymers with 25 and 50 mol % loading of DMODT were tested. As shown in Figure 4d, over a 10 h period, samples using BTH-BTZ photocatalyst showed a greater decrease in peak area over 10 h when compared to the same samples with DiPh-BTZ photocatalyst. The table in Figure 4b shows the numerical values for peak area decrease, comparing all samples analyzed. The use of BTh-BTZ results in (i) an increase in efficiency of irradiation as the λ_max_ of BTh-BTZ is closer to that of the 420 nm irradiation wavelength (Figure 4a) and (ii) an increased irradiation time for ROS production owing to the greater photostability of BTh-BTZ over DiPh-BTZ.

Present in each SEC trace is the gradual emergence of a sharp late dilution peak around 21 min elution time (see Figure S14 of the Supporting Information). This peak was present in all samples after 24 h, even in control samples in the absence of photocatalyst, light, and polymerized DMODT. The nature of this peak was elucidated through control experiments and appears to coincide with both acetone (a byproduct of the polymer chain scission) and very low molecular weight products of the oxidation reaction. The calibration curve for SEC exhibits a sharp drop off in molecular weight after around 21 min, meaning any analyte below this threshold molecular weight will elute at this time.

To conclude, we have shown that a monomer containing selectively oxidizable functionality can be copolymerized with ε-caprolactone. Although our current experimental conditions were unsuccessful in copolymerizations with *rac*-lactide, we are confident that optimization of copolymerization conditions will yield a copolymer with *rac*-lactide and seek to optimize these conditions in future work. Inclusion of the selectively oxidizable functionality at random points along a polymer chain generates sacrificial linkages allowing for the photoinduced accelerated degradation of the polymer in the presence of air and visible light, as measured by continuous SEC analysis. A higher initial loading of the sacrificial linkage in the copolymerization reduces the average molecular weight of the copolymer but also increases the degradation rate of the copolymer chain, with the limiting mol % loading of the sacrificial linkage being 50 mol % in the initial copolymerization feed to effectively produce polymer chains rather than oligomers. Furthermore, we have shown that increasing the absorption efficiency and stability of the photocatalyst used can increase the overall photoinduced degradation of the polymer chain. An initially high photocatalyst loading of 5 mol % was required to ensure degradation on a reasonable time scale, but future efforts could be made to reduce this loading. We found the continuous SEC analysis system described herein to be a versatile and accurate method to follow polymer chain degradation over an extended time period. We foresee that this system would have many applications in the analysis of polymer degradation but also in kinetic studies of polymerizations. We expect that the materials synthesized in this work will become a useful asset in the toolkit of sustainable chemical recycling and a possible additive for biodegradable polymers with the increasing demand for benign-by-design materials.

## Supporting Information Available

Materials and instrumentations, synthesis/preparing methods, characterization, and supplementary figures/tables (PDF)
