# Supplementary material for: A Sacrificial Linker in Biodegradable Polyesters for Accelerated Photoinduced Degradation, Monitored by Continuous Atline SEC Analysis
Source: ACS Macro Lett. 2024 Apr 16;13(5):508–14. doi: 10.1021/acsmacrolett.4c00117 (PMC11112751; doi:10.1021/acsmacrolett.4c00117)
Supplement: Supplementary file 1 — mz4c00117_si_001.pdf [file mz4c00117_si_001.pdf]

Supporting information

A Sacrificial Linker in Biodegradable Polyesters for Accelerated Photoinduced Degradation, Monitored by Continuous Atline SEC Analysis.

*Samuel B. H. Patterson, Valeria Arrighi and Filipe Vilela**

School of Engineering and Physical Sciences, Institute of Chemical Sciences, Heriot-Watt University, Edinburgh, EH14 4AS, UK

*Correspondence to F.Vilela@hw.ac.uk

Table of Contents

1. Experimental 3
1.1 Materials 3
1.2 Instrumentation 3
2 Synthesis of starting materials 4
2.1 2-mercaptoethyl 2-mercaptoacetate 4
2.2 1,4,5-oxadithiepan-2-one (OTP) 5
2.3 5,5-dimethyl-1,4,6-oxadithiocan-2-one (DMODT) 5
2.4 Tetrakis(triphenylphosphine) palladium(0)[4] 7
2.5 4,7-Dibromobenzo[c][1,2,5]thiadiazole (Br_2BTZ) 7
2.6 4,7-diphenylbenzo[c][1,2,5]thiadiazole DiPhBTZ 8
2.7 4,7-Bis(benzo[b]thiophen-2-yl)benzo[c][1,2,5]thiadiazole (BThBTZ) 8
3 Polymerisations 9
3.1 General procedure for co-polymerisation of DMODT and ε-caprolactone 9
3.2 General procedure for co-polymerisation of DMODT and rac-lactide 10
4 Photochemical reactions 10
4.1 Photochemical reactions in batch 10
4.1.1 Photocatalytic oxidation of DMODT monomer 10
4.2 Photochemical reactions in flow 11
5 Characterisation 12
5.1 UV-Vis Spectroscopy 12
5.2 NMR spectra 12
5.2.1 ^{1}H NMR & ^{13}C NMR spectra 12
5.2.2 DOSY 17
5.3 Thermal gravimetric analysis 19
5.4 SEC chromatograms 19
5.5 References 21

1. Experimental

1.1 Materials

All commercially available compounds were purchased from Sigma-Aldrich, Fluorochem, Apollo Scientific or Fischer scientific and used as received, unless otherwise stated. Silica gel for dry vacuum column chromatography was purchased from Sigma-Aldrich as silica gel 60 (0.015-0.040 mm)

1.2 Instrumentation

NMR spectra of synthetic products were recorded using a Bruker AVIII 300 MHz spectrometer using the residual solvent peak as an internal reference. ^{13}C NMR spectra for photocatalysts were recorded using a Bruker AVIIIHD 400 MHz spectrometer. ^{13}C NMR spectra for other synthesised compounds were recorded using a Bruker AVIII 300 MHz spectrometer.

All IR spectra were recorded on solid powder/crystals using a NicoletTMiSTM5 FTIR spectrometer.

UV-Vis absorption spectra for all synthesised photocatalysts were obtained in chloroform solution using a Perkin-Elmer Lambda 35 spectrometer and quartz cuvettes (1 cm pathlength of light). Each set of data was normalised to the maximum absorbance observed in the range of 350 – 800 nm. Emission spectra were also recorded in chloroform solution using a Perkin Elmer LS 55 fluorescence spectrometer using 1 cm quartz cuvettes. The excitation wavelength used was the wavelength of maximum absorption for each individual photocatalyst. All reflections of the excitation wavelengths were removed by subtracting a blank spectrum of chloroform recorded at the same excitation wavelength.

For photocatalytic reactions performed under flow conditions, an easy-Photochem E-Series flow machine was utilised. The reaction mixture was flowed through a coiled section of transparent fluorinated ethylene propylene tubing (total volume 10 mL) irradiated by a Vapourtec UV-150 photochemical reactor equipped with a blue LED module (410 –420 nm, 60W).

For batch photocatalytic reactions, the same lab-built LED modules were employed as in previous work, substituting 410-420 nm LED arrays.[1]

Thermal gravimetric analysis was performed using a Linseis STA PT 1600 instrument on a pre-weighed sample in an alumina crucible under flowing N_2 (BOC oxygen-free nitrogen, $p_{O2} \approx 10^{-5}$ atm, 100 cm^3 min^{-1}). Heat rate: 10°C min^{-1}, Peak temperature: 400°C, Residence time at peak temperature: 10 minutes, Cooling rate: 10°C min^{-1}.

GPC analysis was performed using a Shimadzu High Performance Liquid Chromatograph fitted with a 7.5 mm internal diameter Agilent GPC column. The detector used was a Shimadzu SPD-20A UV-Vis detector set to 254 nm and a Shimadzu RID-20A. HPLC grade tetrahydrofuran (THF, 99.8%, Acros Organics) was utilized as the eluent with flow rate of 1 mL/min with an oven temperature commensurate to the Mark-Houwink parameters used for molecular weight conversion poly(caprolactone): K = 0.01395 α = 0.786 [THF, 25°C], Poly(lactide): K = 0.0549 α = 0.639 [THF, 30°C]). Each measurement was calibrated against 10 polystyrene standards in the range of 162-364,000 g/mol.[2]

2 Synthesis of starting materials

2.1 2-mercaptoethyl 2-mercaptoacetate

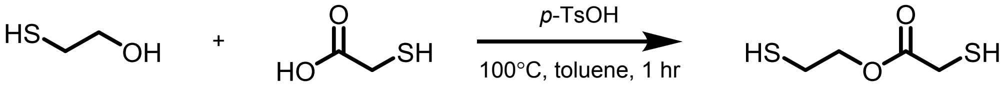

2-mercaptoethyl 2-mercaptoacetate
Molecular Weight: 152.23

Scheme S1: Synthesis of 2-mercaptoethyl 2-mercaptoacetate

2-mercaptoethanol (7.04 mL, 100 mmol 1.00 equiv.), 2-thioglycolic acid (8.33 mL, 120 mmol, 1.20 equiv.) *p*-toluenesulfonic acid (951 mg, 5.00 mmol, 0.05 equiv.) and 300 mL toluene were added to a 500 mL two-neck round bottom flask equipped with a stirrer, and a cooled Dean-Stark trap. The flask was heated to 100°C and a stream of nitrogen was kept flowing over the reaction mixture. The temperature of the mixture was increased from 100°C to 110°C over 4 hours until no more water could be collected in the Dean-Stark trap (theoretical maximum is 1.8 mL). Reaction monitored by TLC (6% MeOH:DCM).

After cooling down the mixture to room temperature, the crude mixture was washed with 100 mL of aq. sodium bicarbonate (distilled water: saturated $NaHCO_3$ in water = 9:1) thrice and then, with 100 mL of distilled water three times. The combined toluene fractions were dried over anhydrous $MgSO_4$, filtered, and concentrated under reduced pressure. The crude product was purified by vacuum distillation (50°C, 0.12 mbar). 8.737 g (57%) of a colourless oil. **^{1}H NMR** ($CDCl_3$, 300 MHz, 25°C): = δ_{ppm} 4.27(t, 2H), 3.29(d, 2H) 2.78 (dt, 2H) 2.03(t, 1H) 1.53(t, 1H) **^{13}C NMR** ($CDCl_3$, 300 MHz, 25 °C): = δ_{ppm} 23.16 (CH_2, SH**C**H_2C=O), 26.28 (CH_2, SH**C**H_2CH_2CO), 66.58 (CH_2, CH_2**C**H_2O), 170.42 (C, **C**=O) **IR:** cm^{-1} 1141 (ester C-O stretch), 1729 (ester C=O stretch), 2564 (thiol S-H stretch), 2950 (alkane CH stretch)

2.2 *1,4,5-oxadithiepan-2-one (OTP)*

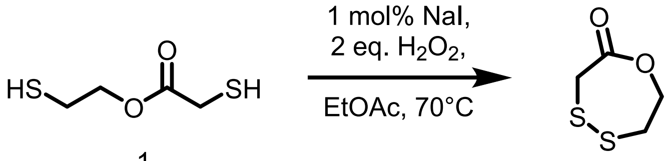

1

1,4,5-oxadithiepan-2-one (OTP)
Molecular Weight: 150.21

Scheme S2: Catalytic oxidative cyclisation of 2-mercaptoethyl 2-mercaptoacetate

A fresh 10 mL solution of sodium iodide (18.74 mg, 12.5x10^{-2} mmol) was prepared in EtOAc. Fresh H_2O_2 was acquired. **1** (190 mg, 1.25 mmol) was dissolved in 25 mL of ethyl acetate. Separately, ethyl acetate (250 mL) was added to a two-neck flask fitted with a reflux condenser and a foil cap for insertion of Teflon tubing. 1.00 mL of the sodium iodide solution and 0.25 ml of hydrogen peroxide solution (30.0~35.5% in water) were then injected into the solution. The mixture was stirred vigorously until the colour of the solution changed to yellow.

Using a syringe pump, the solution of **1** was infused into the sodium iodide solution at 0.042 mL min^{-1} for 10 hours at 70°C using 1 mm ID Teflon tubing. After full infusion, the reaction mixture was cooled to room temperature and washed with 10% aq. sodium thiosulfate (1x 200 mL) and distilled water (2x 250 mL). The organic layer was dried over anhydrous $MgSO_4$, filtered and concentrated under reduced pressure. Product was stored in EtOAc in the dark at 2-8°C Yield: 112.6 mg, (60%)**^{1}H NMR** ($CDCl_3$, 300 MHz, 25°C): = δ_{ppm} 4.66 (t, 2H), 3.93 (s, 2H), 3.09 (br, 2 H) **^{13}C NMR** ($CDCl_3$, 300 MHz, 25°C): = δ_{ppm} 169.11, 69.25, 41.99, 36.75. **IR** $\bar{\nu}$ (cm^{-1}) 3448, 2957, 1729, 1303, 1240, 1099, 1061, 1013, 774, 630, 561, 496, 454, 410

2.3 *5,5-dimethyl-1,4,6-oxadithiocan-2-one (DMODT)*

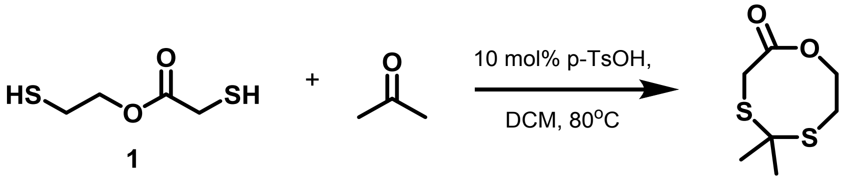

Molecular Weight: 192.29

Scheme S3: Synthesis of 5,5-dimethyl-1,4,6-oxadithiocan-2-one (DMODT)

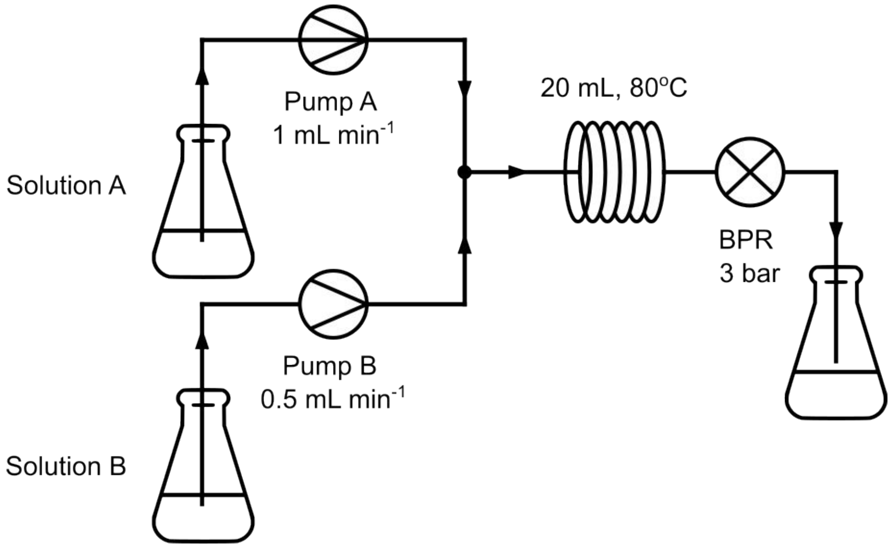

Scheme S4: Flow chemical setup for synthesis of 5,5-dimethyl-1,4,6-oxadithiocan-2-one (DMODT)

Solution A was prepared by adding to a 250 mL RB flask was added *p*-TsOH (1.874g, 10 mol%, 9.85 mmol), acetone (21.9 mL, 295 mmol, 3 equiv.) and DCM (176 mL) (to make a solution 2x the volume of solution B i.e., 200 mL). The flask was then stirred to homogenise. Pump A was primed with this solution. To prepare solution B; **1** (14.998 g, 98.5 mmol, 1 equiv.) was made up to 100 mL with DCM and transferred to an RB flask. Solution B was injected into the flow stream by pump B through a T-junction at 0.5 mL min^{-1}. The combined reaction streams were flowed through a 20 mL reactor coil at 80°C. The flow reaction stream was cooled in a 10 mL coil of tubing submerged in a cold-water bath before being flowed through a back pressure regulator (BPR) at 3 bar pressure. After the reaction was complete (~100 minutes), the reaction mixture was concentrated under reduced pressure and the catalyst was allowed to crystalise before dissolving the crude product in ~150 mL of cold toluene and vacuum filtering. The crude product was then purified by DCVC using DCM : methanol (6% MeOH).[3] 11.559 g (61%) of a colourless, viscous oil. **^{1}H NMR** ($CDCl_3$, 300 MHz, 25°C): = δ_{ppm} 4.27 (t, 2H, C(O)OC$\boldsymbol{H}_2$CH$_2$), 3.42 (s, 2H, C(O)C$\boldsymbol{H}_2$S), 2.27 (t, 2H, C(O)OCH$_2$C$\boldsymbol{H}_2$), 1.62 (s, 6H, SC(C$\boldsymbol{H}_3$)$_2$. **^{13}C NMR** δ_{ppm} = 170.31 (C, ***C***=O), 64.39 (CH$_2$, O***C***H$_2$CH$_2$), 57.03 (C, ***C***(CH$_3$)$_2$), 32.97 (CH$_2$, S***C***C=O), 30.65 (CH$_3$), 28.90 (CH$_2$, OCH$_2$***C***H$_2$). **IR** $\bar{\nu}$ (cm^{-1}) 1107 (ester C-O stretch), 1733 (ester C=O stretch), 2921 (S-C stretch), 2961 (alkane CH stretch)

2.4 Tetrakis(triphenylphosphine) palladium(0)[4]

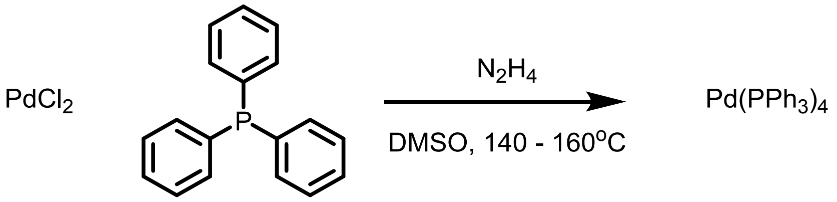

Scheme S5: synthesis of Tetrakis(triphenylphosphine) palladium(0)

A dry 250 mL 2-neck flask was charged with triphenylphosphine (2.63 g, 10 mmol) and palladium (II) chloride (351 mg, 2 mmol) then evacuated and back-filled with nitrogen three times. The flask was then charged with anhydrous DMSO (25 mL) and rapidly evacuated and backfilled with nitrogen three times. The mixture was then heated to 140 –160°C under nitrogen until an orange solution formed, then stirring continued for 15 minutes. Hydrazine hydrate (50 –60%, 0.6 mL) was added then the mixture was removed from the heat and stirring stopped. The mixture was then allowed to cool to room temperature and left to sit for approximately 1 hour. During this time, yellow crystals formed that were then filtered through a sintered glass adaptor under nitrogen. The crystals were then washed with dry methanol (5 x 15 mL) and dry diethyl ether (5 x 10 mL) then allowed to dry under nitrogen and vacuum. The crystals were then transferred to a dry round bottom flask, sealed with a septum then evacuated and backfilled with nitrogen five times. The final mass of dried crystals was 2.057 g (89%).

2.5 4,7-Dibromobenzo[c][1,2,5]thiadiazole (Br_2BTZ)

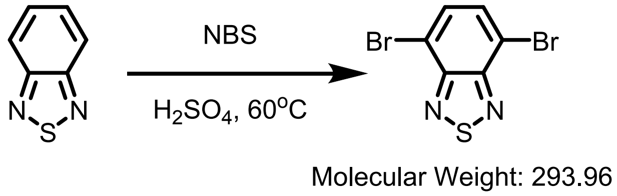

Scheme S6: Synthesis of 4,7-Dibromobenzo[c][1,2,5]thiadiazole (Br_2BTZ)

Benzo[c][1,2,5]thiadiazole(2.5 g, 18.4 mmol) and N-bromosuccinimide (NBS) (6.877 g, 38.64 mmol) were dissolved in concentrated sulfuric acid (25 mL) and the mixture heated to 60°C for 4 hours. Following this, the reaction was allowed to cool to room temperature and then poured onto an ice-water mixture. The precipitate that formed was then filtered and washed with copious amounts of water. The final product was dried under vacuum to give an off-white powder (4.524 g, 85%) **^{1}H NMR** (CDCl3, 300 MHz, 25.0°C): = δ_{ppm} 7.73 (s, 2 H). **^{13}C NMR** (CDCl3, 400 MHz, 25.0°C): = δ_{ppm} 153.0 (C), 132.3 (CH), 113.9 (C).

2.6 4,7-diphenylbenzo[c][1,2,5]thiadiazole DiPhBTZ

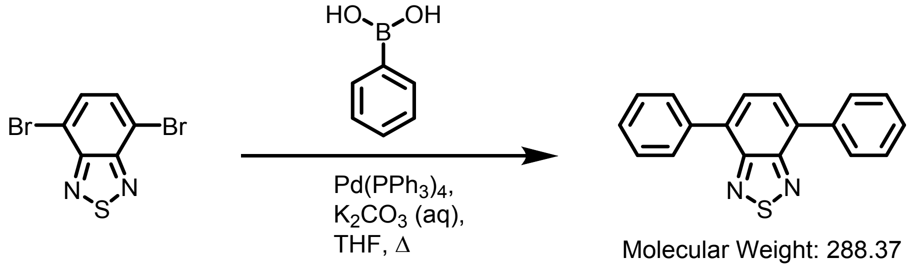

Scheme S7: Synthesis of 4,7-diphenylbenzo[c][1,2,5]thiadiazole DiPhBTZ

A dry 2-neck flask was charged with benzene boronic acid (1.9163 g, 15.72 mmol), 4,7-dibromo-2,1,3-benzothiadiazole (2.100 g, 7.14 mmol), potassium carbonate (2.3695 g, 17.15 mmol), $Pd(PPh_3)_4$ (58 mg, 0.05 mmol). The flask was connected to a Schlenk line then degassed and backfilled with nitrogen three times. Degassed THF (40 mL) and degassed deionised water (5 mL) were added, and the reaction heated to 70°C for 16 hours. Following this the mixture was poured onto water (50 mL) and extracted with DCM (3 x 35 mL). The combined organic phases were washed with deionised water (50 mL), dried over $MgSO_4$ and the solvent removed under reduced pressure. The crude product was then recrystalised from hot methylated spirits. Yield: 1.5501 g, 34% of yellow crystals **^{1}H NMR** ($CDCl_3$, 300 MHz, 25°C): δ_{ppm} 7.97 (m, 4H), 7.79 (s, 2H), 7.56 (m, 4H), 7.47 (m, 2H) **IR** $\bar{\nu}$ (cm^{-1}) 3060 (w, C-H str.). **UV-VIS** ($CHCl_3$) λ_{max} (nm) 380.

2.7 4,7-Bis(benzo[b]thiophen-2-yl)benzo[c][1,2,5]thiadiazole (BThBTZ)

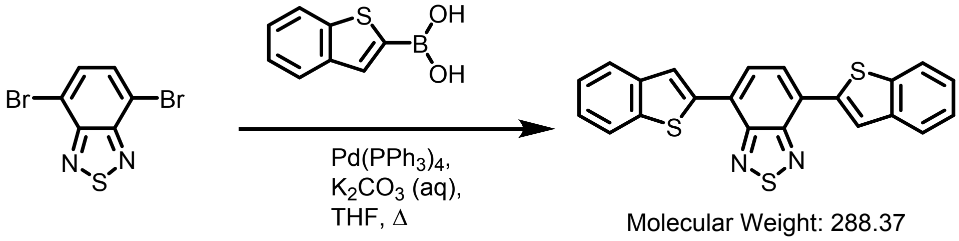

Scheme S8: Synthesis of 4,7-Bis(benzo[b]thiophen-2-yl)benzo[c][1,2,5]thiadiazole (BThBTZ)

General procedure A for Suzuki-Miyaura coupling as above using 2-benzo[b]thiophen-2-ylboronic acid (445 mg, 2.5 mmol), 4,7-dibromobenzo[c][1,2,5]thiadiazole(294 mg, 1.0 mmol), potassium carbonate (276 mg, 2.0 mmol) and $Pd(PPh_3)_4$ (58 mg, 0.05 mmol). Crude product was washed with hot methylated spirits to give a red powder (273 mg, 69%).**^{1}H NMR**(CDCl3, 300 MHz, 25.0°C) δ_{ppm} 8.59 (d, 2 H), 7.99 (s, 2H), 7.90 (m, 4 H), 7.39 (m, 4 H). **^{13}C NMR** (CDCl3, 75.5 MHz, 25.0°C) δ_{ppm} 152.9 (C), 140.6 (C), 139.7 (C), 127.1 (CH), 126.7 (C), 125.5 (CH), 125.3 (CH), 124.7 (CH), 124.4 (CH), 122.1 (CH). **IR** ν (cm^{-1}) 3046 (w, C-H str.) 3060 (w, C-H str.). **UV-VIS** ($CHCl_3$) λ_{max} (nm) 540.

3 Polymerisations

3.1 General procedure for co-polymerisation of DMODT and ε-caprolactone

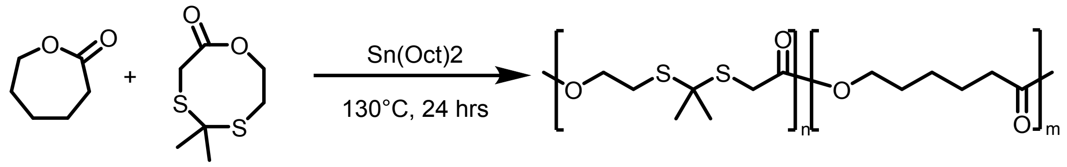

Scheme S9: Random co-polymerisation of ε-caprolactone and DMODT in solution

Glassware was flame-dried before use. ε-caprolactone (ε-CL) was dried over CaH_2 and distilled before use*. Tin octanoate ($Sn(Oct)_2$) was dissolved in anhydrous toluene (0.1 M) and kept in the dark. Anhydrous benzyl alcohol was dissolved in anhydrous toluene (0.1 M) and kept in the dark. A 1.2 M solution of DMODT was prepared in anhydrous toluene. For polymerisations, the total number of moles (with respect to the two monomers) was fixed at 10.0 mmol. Benzyl alcohol was used as the initiator and the monomer to initiator to catalyst ratio was fixed at 100:1:0.5.[5] A typical procedure was followed: DMODT solution (1.2 M, 0.412 mL, 0.50), ε-CL (1.05 mL, 9.50 mmol) were added sequentially to a N_2 purged polymerisation tube, under a blanket of N_2. The reagents were stirred for 10 minutes until homogenous. $Sn(Oct)_2$ solution (50 μL, 5.00 μmol) and benzyl alcohol (100 μL, 10.0 μmol) solutions were then added under a N_2 blanket along with anhydrous toluene (3 mL). Toluene was then removed by azeotropic distillation under vacuum. An additional 4 mL of anhydrous toluene was added to each sample before transferring to 10 mL pressure sealed polymerisation vials in a lab-made aluminium heating block, preheated to 80°C under a blanket of N_2. The reaction was then heated to 130°C for 24 hrs under N_2. The reaction was stopped by cooling to room temperature. Toluene was removed from the mixture under vacuum and the mixture was then dissolved in chloroform (~10 mL) and precipitated into ice cold diethyl ether (10x volume) for 2 hrs at -20°C before filtering under gravity and drying at room temperature at 12 mbar for 24 hours. Dissolution and precipitation was repeated as above 2 more times to purify the polymer. For 50 mol% DMODT sample: **^{1}H NMR**(CDCl3, 300 MHz, 25.0°C) δ_{ppm} 4.27 (t, 2H), 4.06 (t, 2H), 3.43 (s, 2H), 2.88 (t, 2H) 2.31 (t, 2H). 1.64 (m, 10H) 1.38 (m, 2H). **^{13}C NMR** (CDCl3, 300 MHz, 25.0°C) δ_{ppm} 173.55 (C), 170.22 (C), 64.42 (CH_2), 64.16 (CH_2), 57.05 (C), 34.15 (CH_2), 33.02 (CH_2), 30.65 (CH_3), 28.90 (CH_2), 28.33 (CH), 25.57 (CH_2), 24.60 (CH_2).

*A dry single neck round bottom flask equipped with stirrer bar was put under vacuum and gently heated with a heat gun and evacuated with Nitrogen three times. 50 mL of ε-CL was added under

a blanket of N_2 followed by CaH_2 (1/2 water content of reagent). Mixture stirred for 48 hours under N_2. Absence of water was confirmed by ^1H NMR analysis. The ε-CL/BuOH was then distilled under vacuum and kept under nitrogen with 1.4 Å molecular sieves until further use.

3.2 General procedure for co-polymerisation of DMODT and rac-lactide

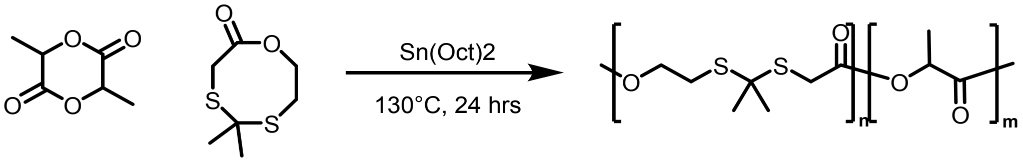

Scheme S10: Random co-polymerisation of *rac*-lactide and DMODT in the melt

Glassware was flame-dried before use. Tin octanoate ($Sn(Oct)_2$) was dissolved in anhydrous toluene (0.1 M) and kept in the dark. Benzyl alcohol was dissolved in anhydrous toluene (0.1 M) and kept in the dark. A 1.20 M solution of DMODT in anhydrous toluene was prepared and made up to 100 mL. *Rac*-Lactide was triply sublimed and stored at -20°C before use. For polymerisations, the total number of moles (with respect to the two monomers) was fixed at 10.0 mmol. Benzyl alcohol was used as the initiator and the monomer to initiator to catalyst ratio was fixed at 100:1:0.5.[5] *Rac*-lactide (855.94 mg, 7.50 mmol) was added to an N_2 purged 50 mL RBF with stirrer bar under a blanket of N_2. A 3-way gas tap was fitted, and the RBF was then evacuated and backfilled with N_2 three times. After this, DMODT solution (2.079 mL, 2.50 mmol), Benzyl alcohol solution (100 µL, 0.01 mmol) and $Sn(Oct)_2$ Solution (50 µL, 0.005 mmol) were injected through the gas tap under a flow of N_2. The mixture was stirred to homogenise for 10 minutes. Toluene was then removed by azeotropic distillation under vacuum. The vials were then heated to 130 °C in a silicone oil bath for a melt polymerisation lasting 24 hrs. The reaction was stopped by cooling to room temperature. The crude mixture was then dissolved in chloroform (~10 mL) and precipitated into ice cold methanol (10x volume acidified with 3 drops 3 M HCl) and precipitated for 2 hrs at -20°C before filtering under gravity and drying under vacuum at 40°C. For 25mol% DMODT sample: **^{1}H NMR**(CDCl3, 300 MHz, 25.0 °C) δ_{ppm} 5.17 (m, 1H), 4.27 (t, 2H), 3.43 (s, 2H), 2.88 (t, 2h), 1.58 (m, 8H). **^{13}C NMR** (CDCl3, 300 MHz, 25.0 °C) δ_{ppm} 170.6 (C), 169.3 (C), 68.9 (CH), 64.2 (CH_2), 57.0 (C), 32.9 (CH_2), 30.6 (CH_3), 28.7 (CH_2), 16.5 (CH_3).

4 Photochemical reactions

4.1 Photochemical reactions in batch

4.1.1 Photocatalytic oxidation of DMODT monomer

A solution of DMODT at 0.2 mmol mL^{-1} (384.54 mg, 2 mmol) was prepared and made up to 10 mL in $CDCl_3$, which was then bubbled with oxygen for 10 minutes. A separate solution of

DiPhBTZ at 0.05 mmol mL^{-1} (144.2 mg, 0.5 mmol) was also prepared and made up to 10 mL with $CDCl_3$. Each sample was prepared in a NMR tube by transferring 0.1 mL of BTZ (5 μmol, 5mol% loading wrt DMODT) solution and 0.5 mL of DMODT solution (0.1 mmol) into the tubes. 10 samples were prepared in total. The samples were then suspended at a fixed distance of 7 cm from the 410-420 nm LED array in a lab-made photoreactor consisting of a mirrored box. The samples were removed sequentially at regular time intervals over the course of 6 hours. The final sample was analysed by ^{1}H NMR and then returned to the photoreactor overnight. The first sample (t_0) was analysed by ^{1}H NMR and then kept shielded from light through the course of the experiment and sampled at the same time intervals as the other samples as a negative control sample. Other control samples in the absence of oxygen and photocatalyst were also performed as above.

4.2 Photochemical reactions in flow

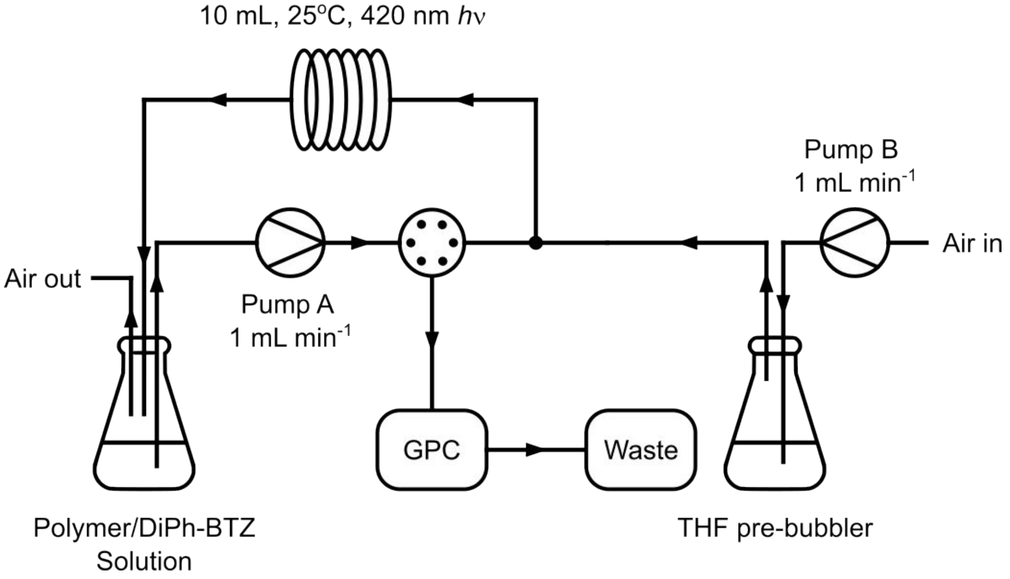

Scheme S11: Flow diagram for photochemical reactions performed in flow

Poly(DMODT-co-CL) (125 mg) was dissolved in 25 mL of HPLC grade THF in a polymerisation tube fitted with a magnetic stirrer bar and a rubber septum to form a 0.5wt% solution, (minimum conc. required for GPC analysis). A 0.1 M standard solution of DiPhBTZ in HPLC grade THF was prepared, made up to 5 mL (144.2 mg DiPhBTZ). 43 μL (0.1wt% wrt polymer) of the DiPhBTZ solution was injected into the polymer solution (negligible dilution effect). The mixture was then stirred for 10 mins to homogenise. An inline filter was plumbed through the rubber septum into the polymer/DiPhBTZ solution. This solution was then pumped at 1 mL min^{-1} (pump A) through a 20 μL sample loop on a 2-position, 6-port valve. The sample loop fed back into a T-junction to be mixed with air being pumped at 1 mL min^{-1} (pump C). The air was

saturated with THF vapour *via* a pre-bubbler to minimise solvent evaporation. The mixed phases were then flowed through a 10 mL reactor coil irradiated with 420 nm light before being recycled into the polymer/DiPhBTZ solution. A blead valve was incorporated into the rubber septum to allow air to escape from the polymer/DiPhBTZ solution. At programmed intervals, the valve switched from the default to active position to run an aliquot of the reaction solution through at-line GPC. The reaction was allowed to proceed for 24 hrs with a 30-minute sampling window.

5 Characterisation

5.1 UV-Vis Spectroscopy

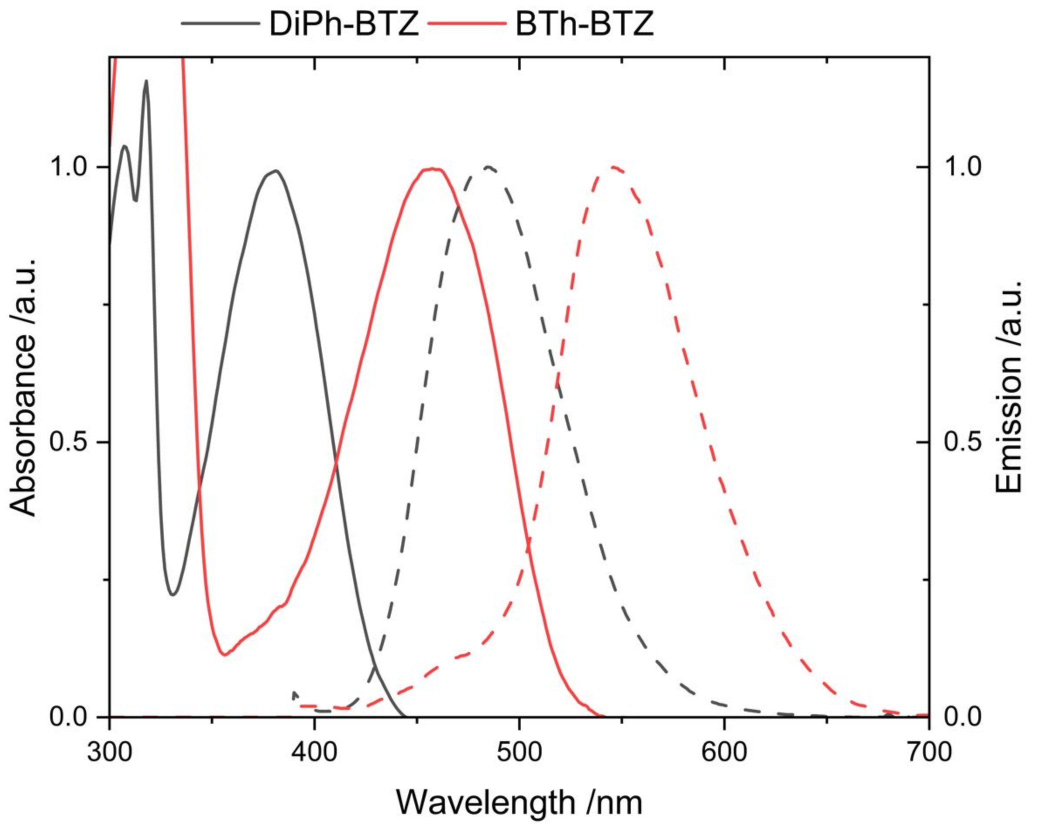

Figure S1: Normalised absorption (solid lines) and emission (dotted lines) of the photocatalysts

5.2 NMR spectra

5.2.1 <u>^{1}H NMR & ^{13}C NMR spectra</u>

All spectra for photocatalysts and their starting materials can be found in previous work.[4]

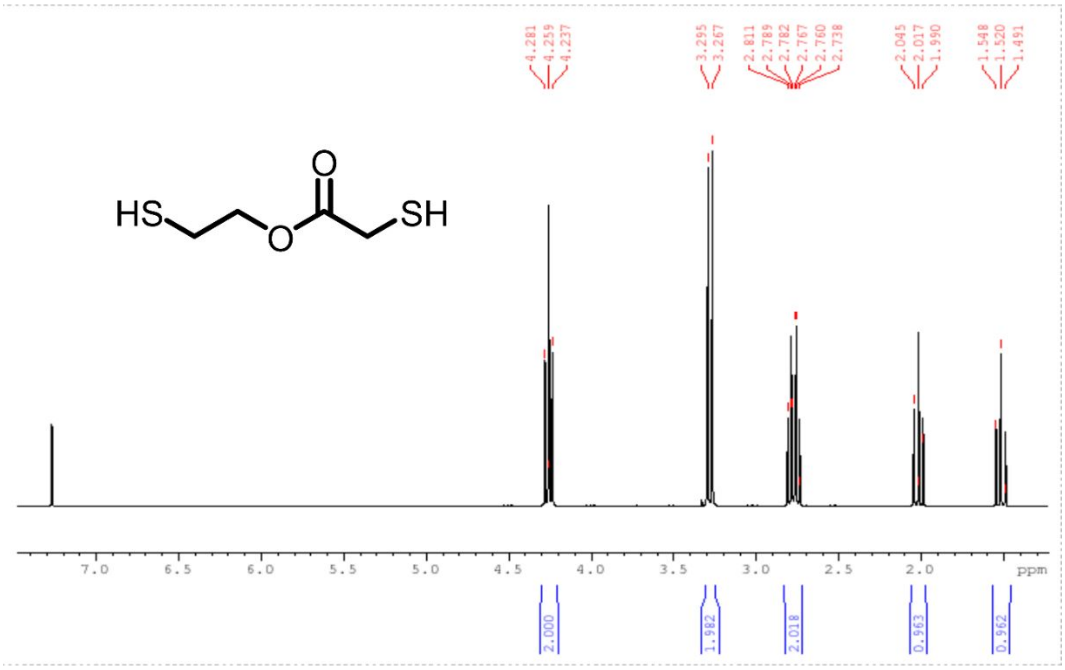

Figure S2: ^1H NMR of 2-mercaptoethyl 2-mercaptoacetate (solvent residues: $CHCl_3$ = 7.26 ppm)

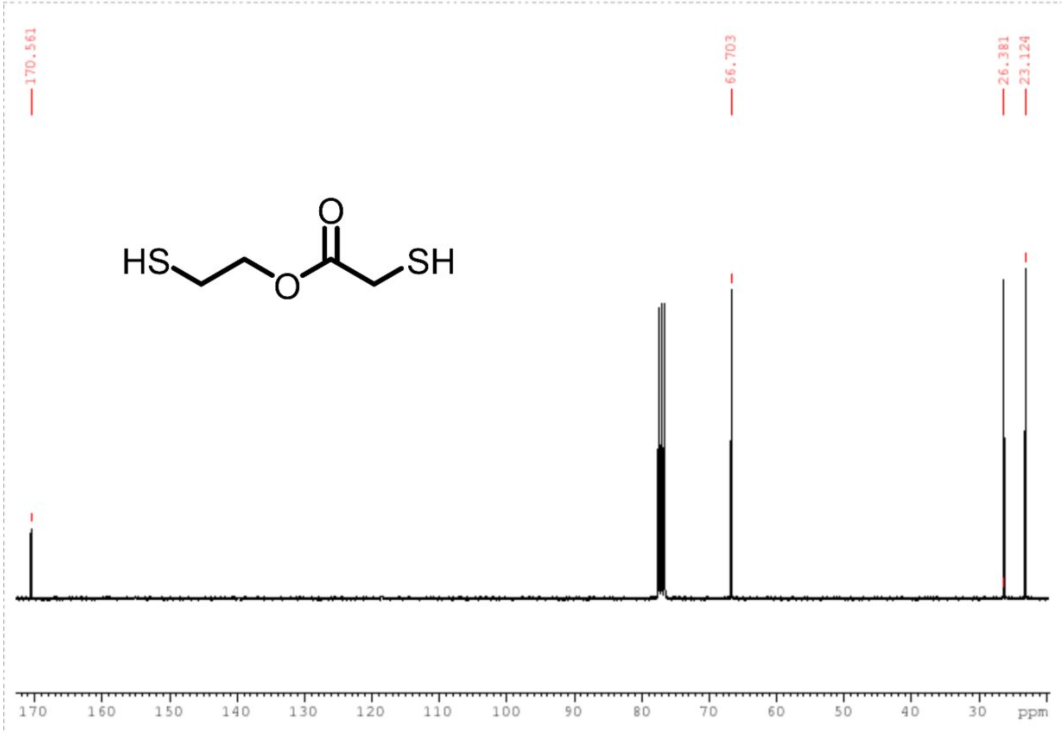

Figure S3: ^{13}C NMR of 2-mercaptoethyl 2-mercaptoacetate

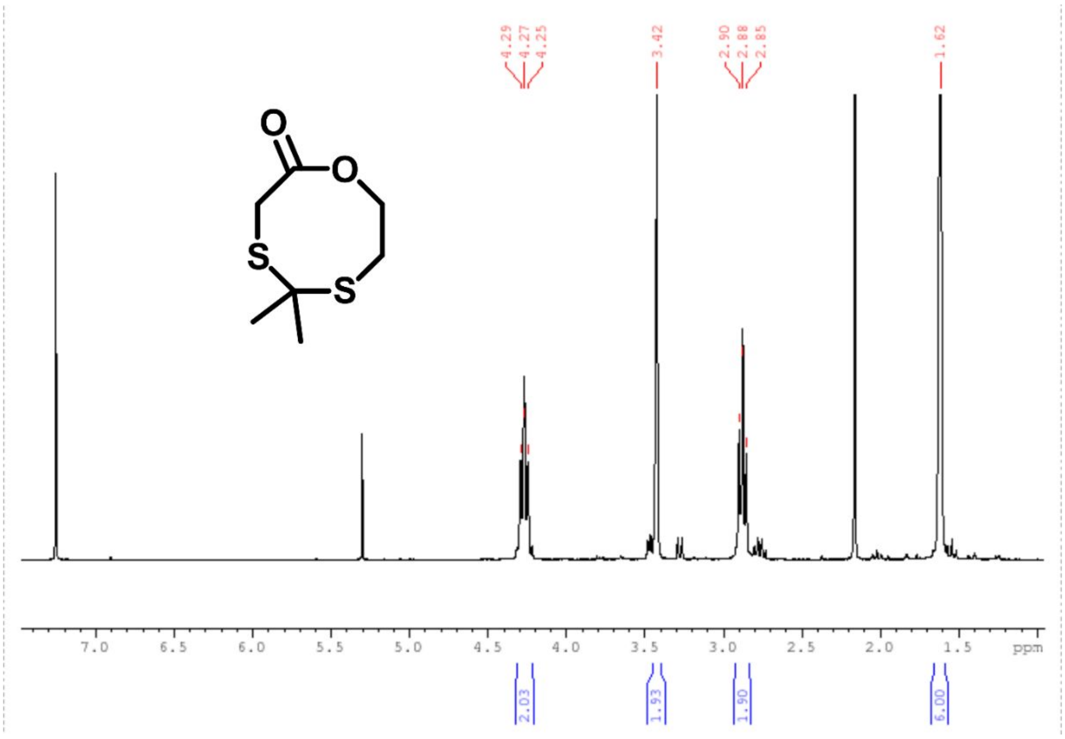

Figure S4: ^{1}H NMR of DMODT (solvent residues: $CHCl_3$ = 7.26 ppm, DCM = 5.30 ppm, acetone = 2.17 ppm)

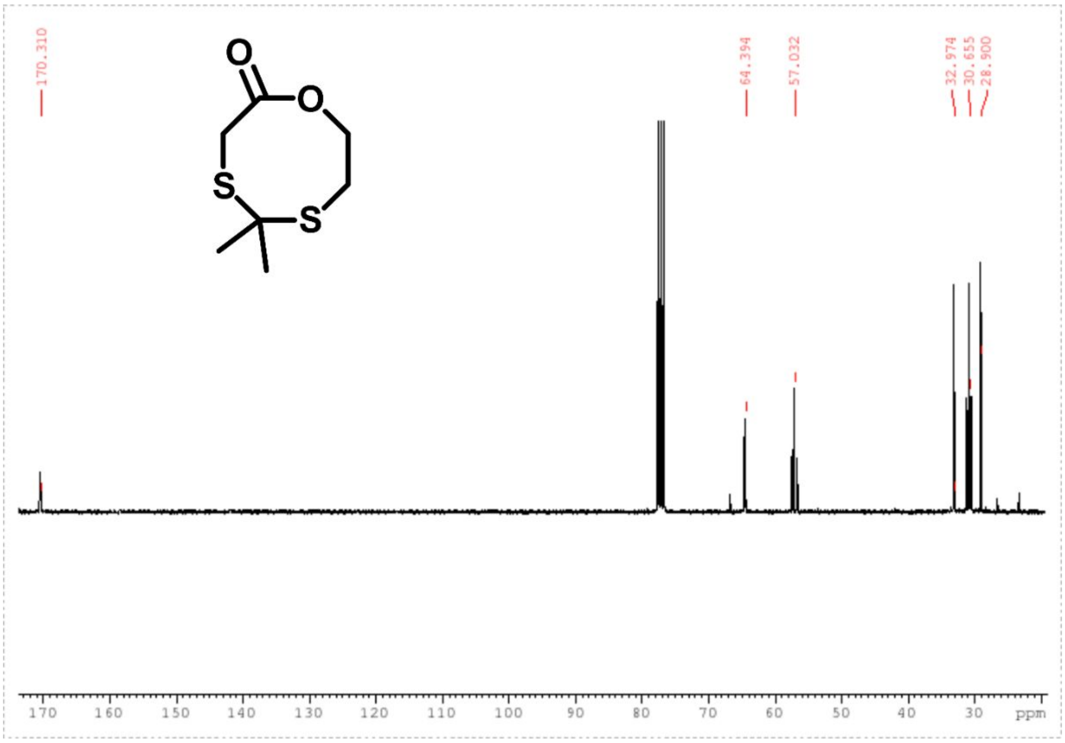

Figure S5: ^{13}C NMR of DMODT

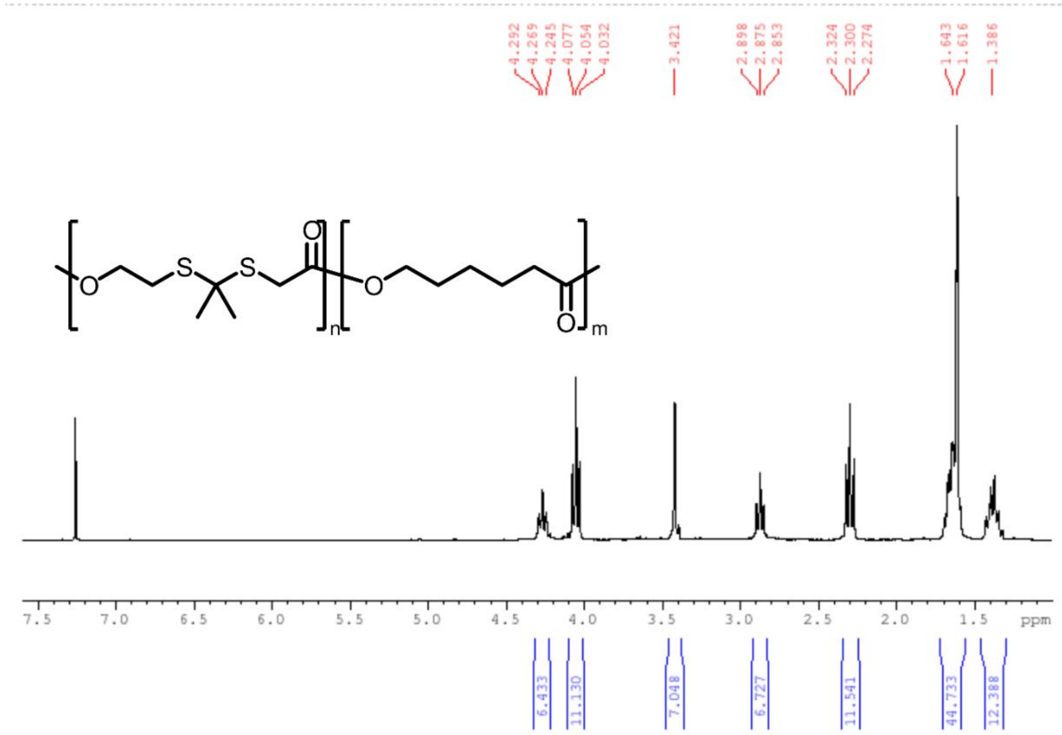

Figure S6: ^{1}H NMR of poly(DMODT)-co-caprolactone (50mol% DMODT) (solvent residues: $CHCl_3$ = 7.26 ppm)

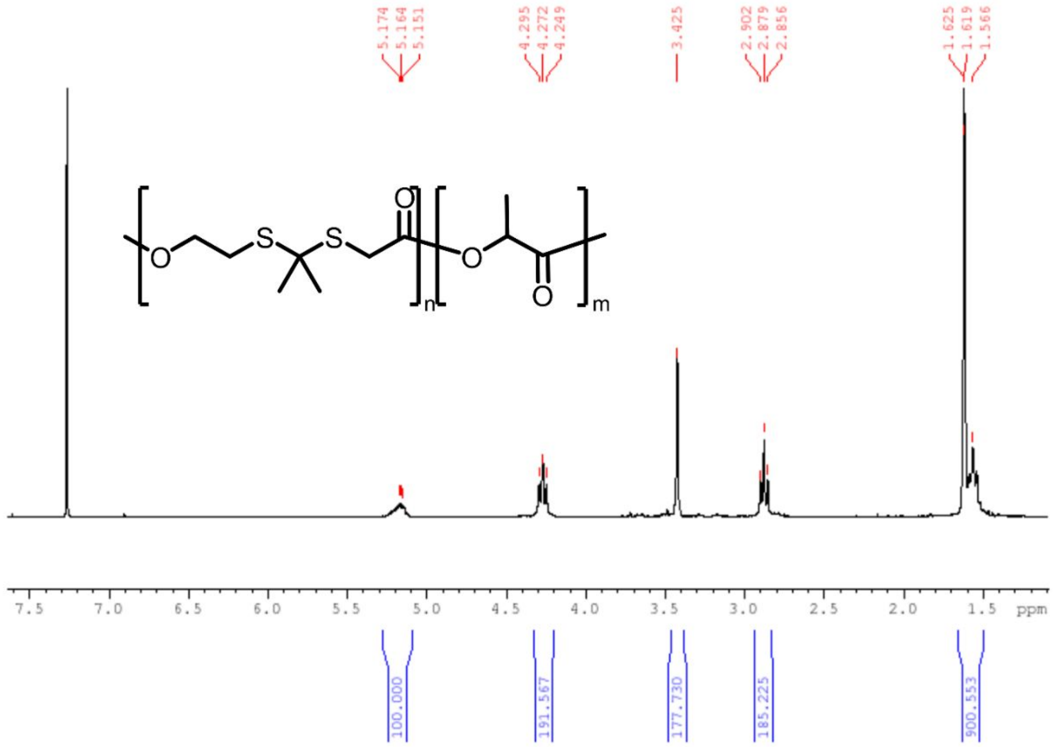

Figure S7: ^{1}H NMR of poly(DMODT)-co-lactide (50mol% DMODT) (solvent residues: $CHCl_3$ = 7.26 ppm)

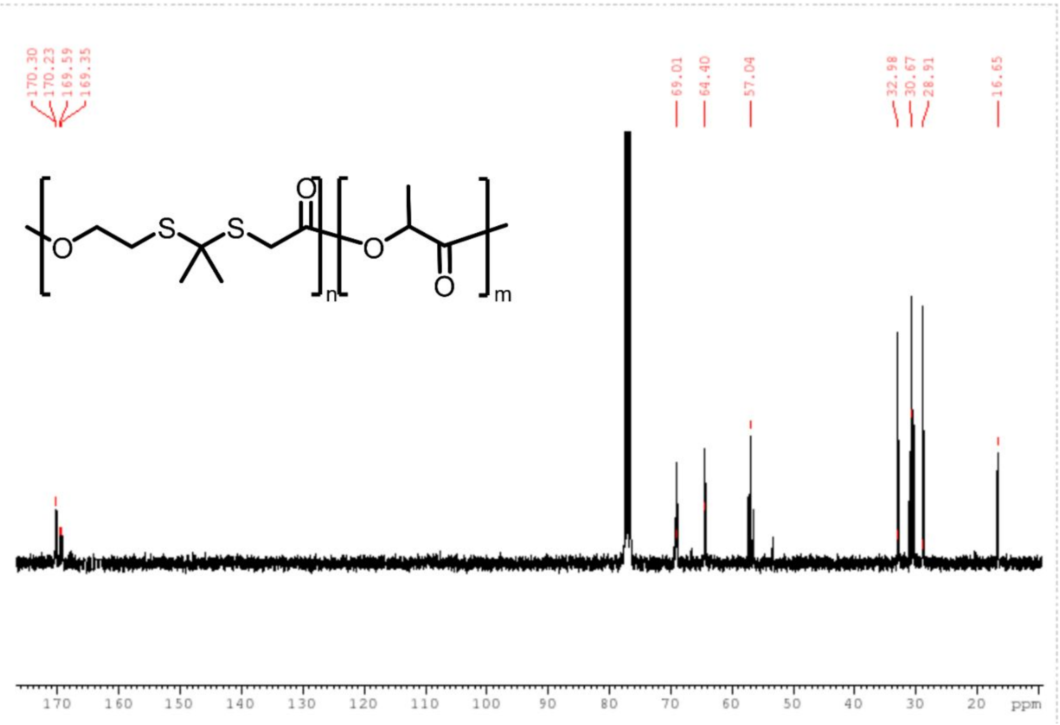

Figure S8: ^{13}C NMR of poly(DMODT)-co-lactide (50 mol% DMODT)

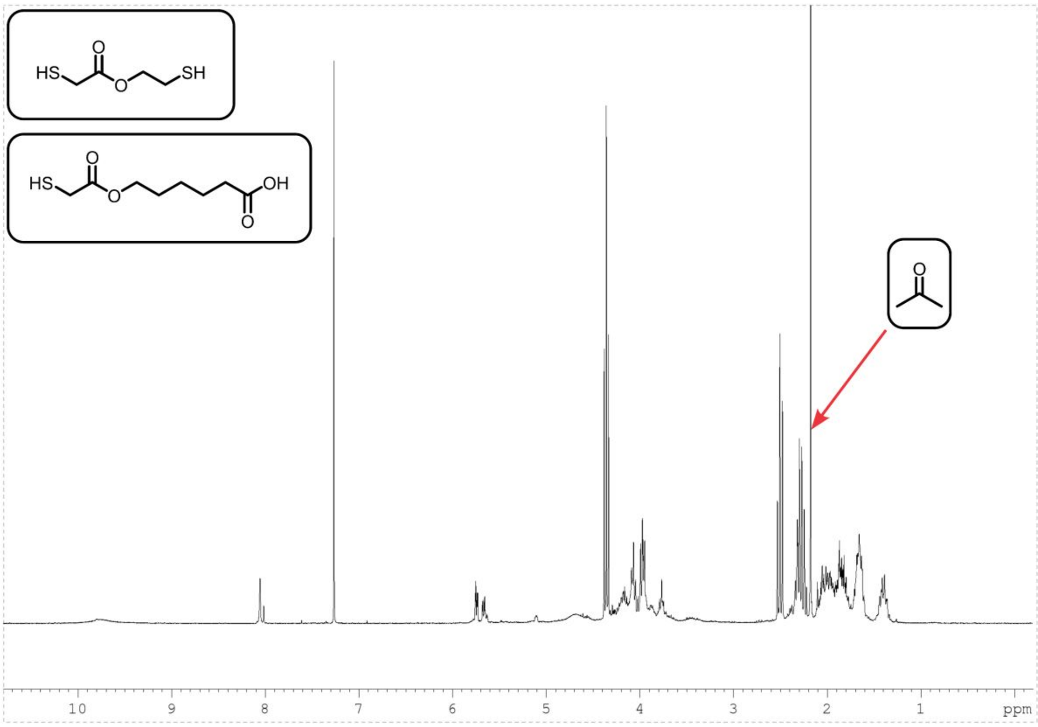

Figure S9: ^{1}H NMR of the residue left from photocatalytic degradation of poly(DMODT-co-caprolactone) (50 mol% DMODT) showing potential degradation products and highlighting the presence of an acetone singlet at 2.17 ppm

5.2.2 DOSY

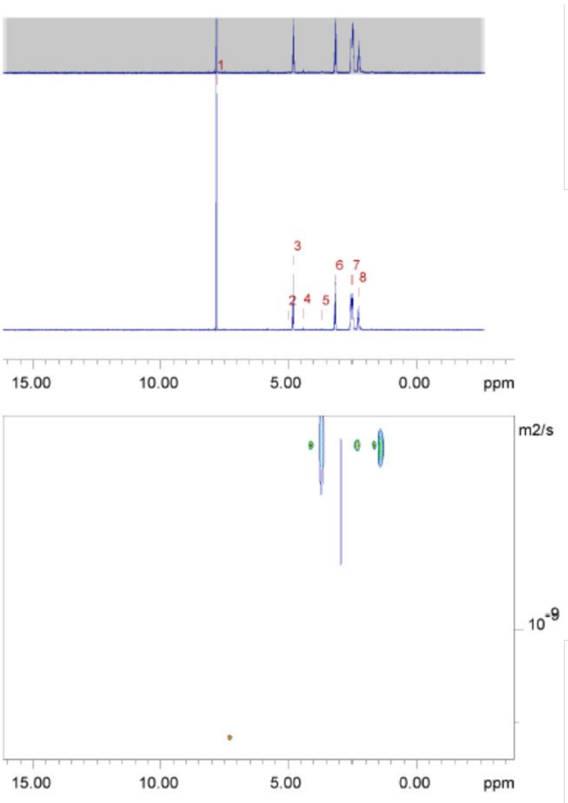

5mol% poly(DMODT-co-caprolactone

Fitted function:	f (x) = Io * exp (-D * x^2 * gamma^2 * littleDelta^2 (bigDelta-littleDelta/3)* 10^4
used gamma:	26752 rad/(s*Gauss)
used little delta:	0.0020000 s
used big delta:	0.049900 s
used gradient strength:	variable
Random error estimation of data:	RMS per spectrum (or trace/plane)
Systematic error estimation of data:	worst case per peak scenario
Fit parameter Error estimation method:	from fit using arbitray y uncertainties
Confidence level:	95%
Used peaks:	peaks from C:/Bruker/TopSpin3.6.4/data/SBP/nmr/SBPDOSY214 P5%/2/pdata/1/peaklist1D.xml
Used integrals:	peak intensities
Used Gradient strength:	all values (including replicates) used

Peak name	F2 [ppm]	D [m2/s]	error	fitInfo
1	7.285	2.23e-09	6.843e-12	Done
2	4.293	0.00	0.000	Fail
3	4.087	2.52e-10	4.937e-12	Done
4	3.675	2.48e-10	9.429e-11	Done
5	2.913	3.89e-10	3.015e-10	Done
6	2.333	2.52e-10	5.322e-12	Done
7	1.679	2.55e-10	5.123e-12	Done
8	1.413	2.58e-10	2.355e-11	Done

Figure S10: DOSY NMR for 5mol% DMODT poly(DMODT-co-caprolactone)

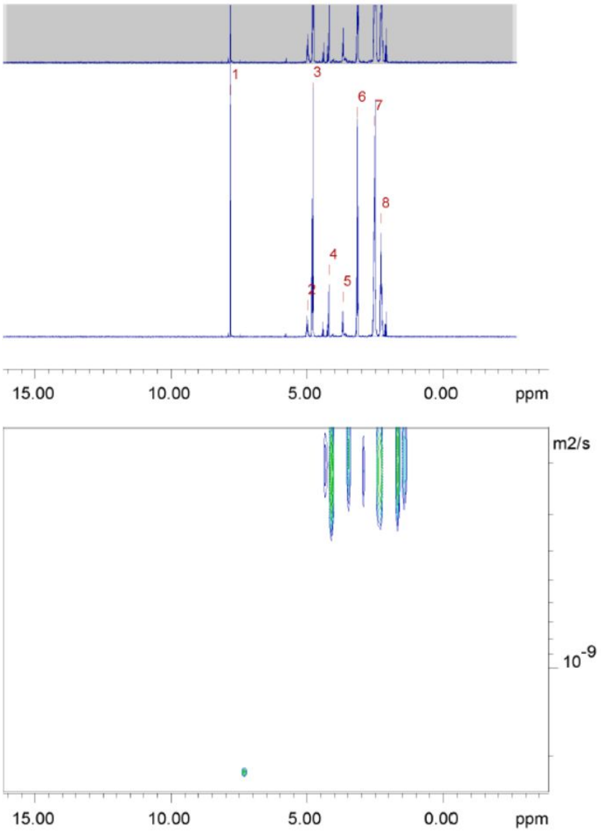

25mol% poly(DMODT-co-caprolactone

Fitted function:	f (x) = Io * exp (-D * x^2 * gamma^2 * littleDelta^2 (bigDelta-littleDelta/3)* 10^4
used gamma:	26752 rad/(s*Gauss)
used little delta:	0.0020000 s
used big delta:	0.049900 s
used gradient strength:	variable
Random error estimation of data:	RMS per spectrum (or trace/plane)
Systematic error estimation of data:	worst case per peak scenario
Fit parameter Error estimation method:	from fit using arbitray y uncertainties
Confidence level:	95%
Used peaks:	peaks from C:/Bruker/TopSpin3.6.4/data/SBP/nmr/SBPDOSY214 P25%/2/pdata/1/peaklist1D.xml
Used integrals:	peak intensities
Used Gradient strength:	all values (including replicates) used

Peak name	F2 [ppm]	D [m2/s]	error	fitInfo
1	7.287	2.26e-09	4.558e-11	Done
2	4.303	2.04e-10	6.115e-11	Done
3	4.087	2.16e-10	6.386e-11	Done
4	3.453	1.87e-10	6.336e-11	Done
5	2.909	2.14e-10	6.679e-11	Done
6	2.333	2.14e-10	5.910e-11	Done
7	1.673	1.94e-10	6.531e-11	Done
8	1.413	1.97e-10	6.236e-11	Done

Figure S11: DOSY NMR for 25mol% DMODT poly(DMODT-co-caprolactone)

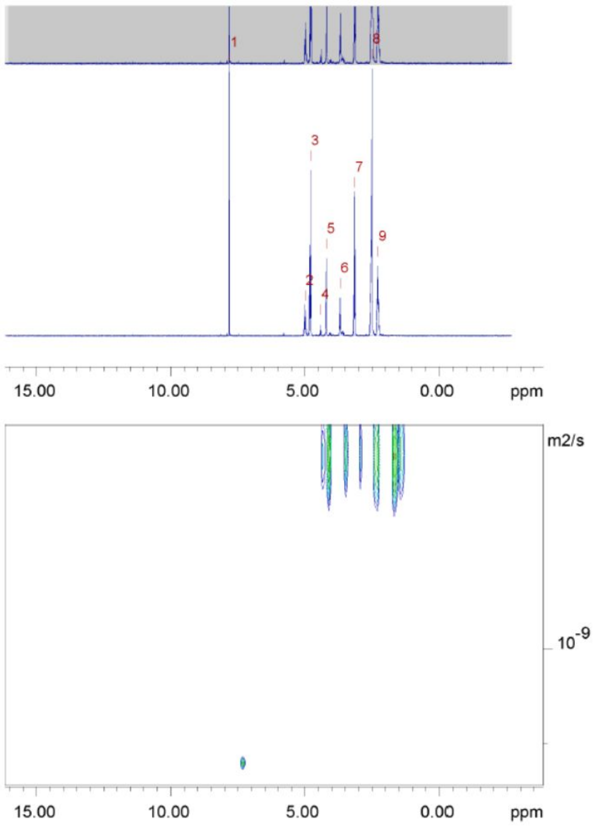

50mol% poly(DMODT-co-caprolactone

Fitted function:	f (x) = Io * exp (-D * x^2 * gamma^2 * littleDelta^2 (bigDelta-littleDelta/3)* 10^4
used gamma:	26752 rad/(s*Gauss)
used little delta:	0.0020000 s
used big delta:	0.049900 s
used gradient strength:	variable
Random error estimation of data:	RMS per spectrum (or trace/plane)
Systematic error estimation of data:	worst case per peak scenario
Fit parameter Error estimation method:	from fit using arbitray y uncertainties
Confidence level:	95%
Used peaks:	peaks from C:/Bruker/TopSpin3.6.4/data/SBP/nmr/SBPDOSY214 P50%/3/pdata/1/peaklist1D.xml
Used integrals:	peak intensities
Used Gradient strength:	all values (including replicates) used

Peak name	F2 [ppm]	D [m2/s]	error	fitInfo
1	7.287	2.33e-09	6.877e-11	Done
2	4.302	2.44e-10	6.075e-11	Done
3	4.087	2.36e-10	5.940e-11	Done
4	3.675	2.82e-10	6.362e-11	Done
5	3.453	2.37e-10	5.358e-11	Done
6	2.909	2.38e-10	6.146e-11	Done
7	2.331	2.40e-10	5.928e-11	Done
8	1.647	2.42e-10	5.871e-11	Done
9	1.407	2.38e-10	5.925e-11	Done

Figure S12: DOSY NMR for 50mol% DMODT poly(DMODT-co-caprolactone)

5.3 Thermal gravimetric analysis

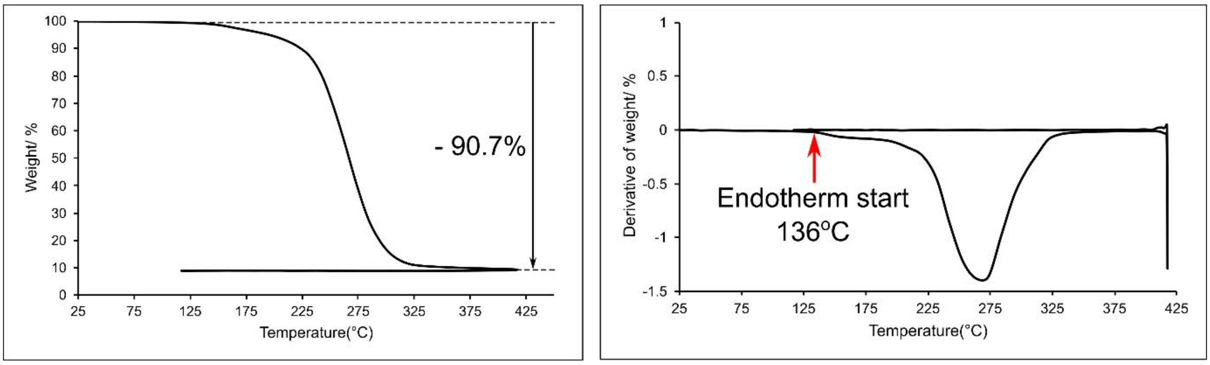

Figure S13: Thermal gravimetric analysis of DMODT monomer showing the onset of thermal degradation at 136°C.

5.4 SEC chromatograms

SEC chromatograms of poly(DMODT-co-caprolactone) are available in the main text, Figure 2.

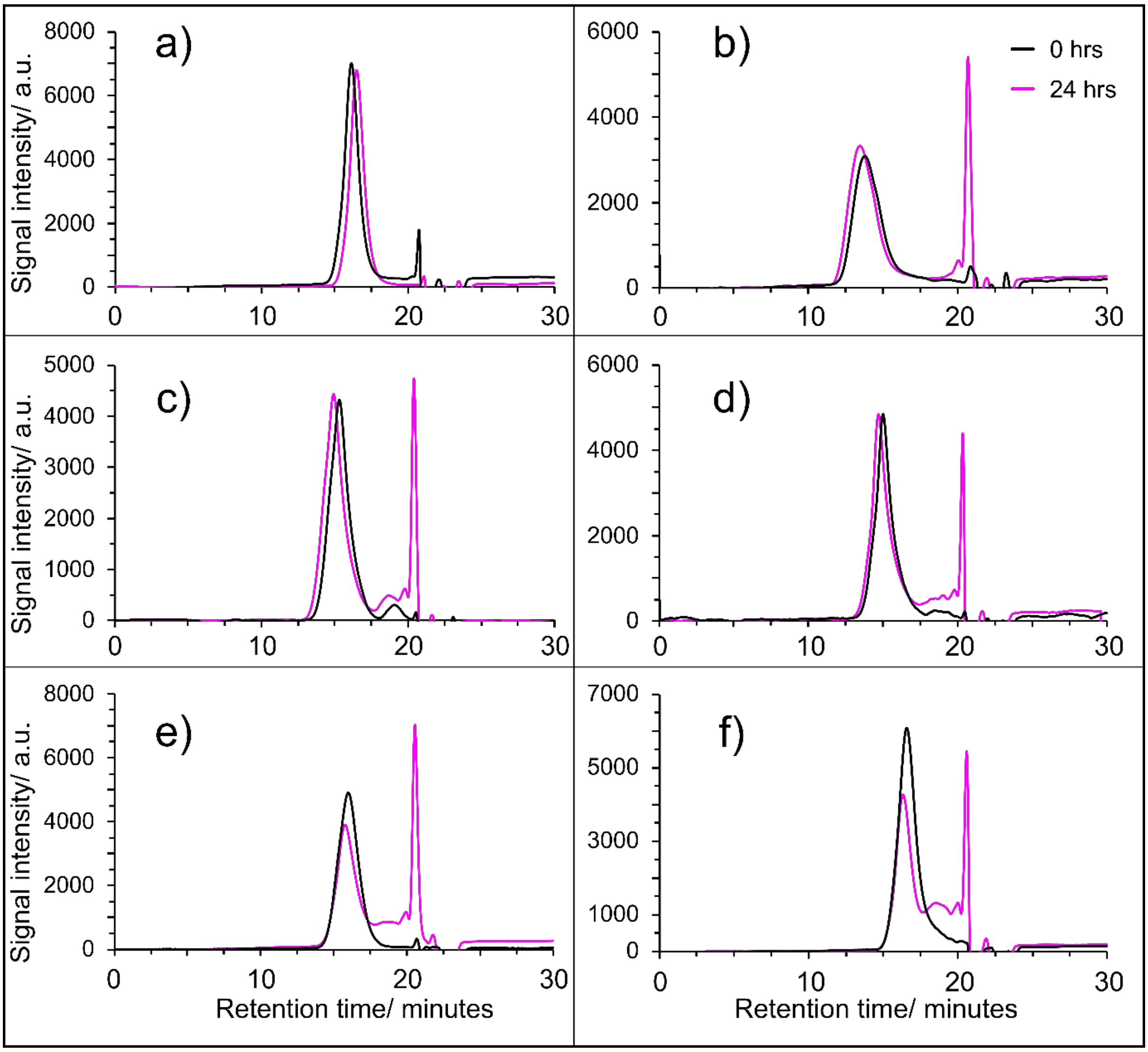

Figure S14 SEC traces of poly(DMODT-co-caprolactone) with varying amount of initial DMODT monomer before and after undergoing irradiation of 420 nm for 24 hours with DiPh-BTZ photocatalyst; (a) no photocatalyst, (b) 0mol% DMODT, (c) 5 mol% DMODT, (d) 10mol% DMODT, e) 25mol% DMODT, f) 50mol% DMODT

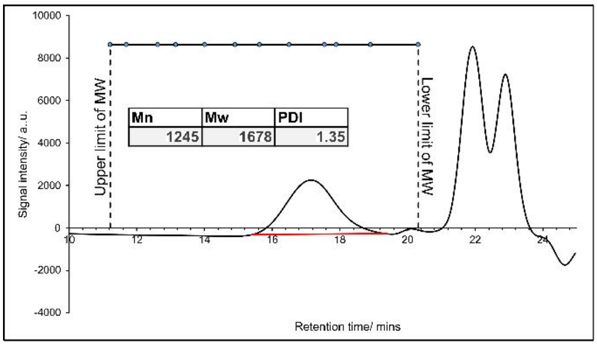

0mol% DMODT i.e. PLA

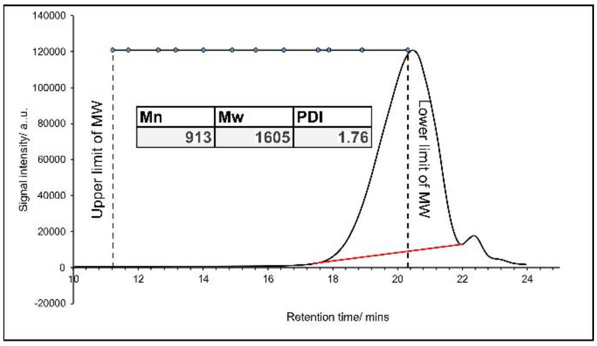

25mol% DMODT poly(DMODT)-co-PLA

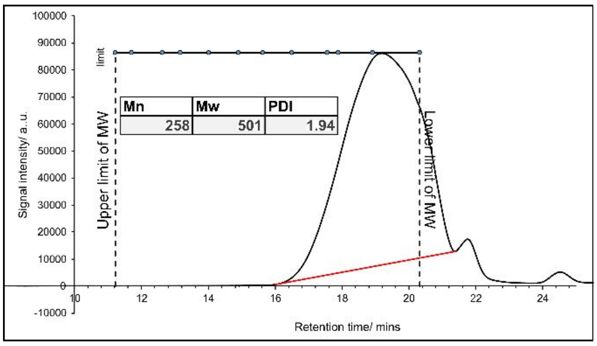

50mol% DMODT poly(DMODT)-co-PLA

Figure S15: SEC traces of unsuccessful co-polymerisations of DMODT and *rac*-lactide

5.5 References

1. Thomson CG, Jones CMS, Rosair G, Ellis D, Marques-Hueso J, Lee A-L, Vilela F (2020) Continuous-flow synthesis and application of polymer-supported BODIPY Photosensitisers for the generation of singlet oxygen; process optimised by in-line NMR spectroscopy. J Flow Chem 10:327–345. https://doi.org/10.1007/s41981-019-00067-4

2. Jenkins DT, Fazekas E, Patterson SBH, Rosair GM, Vilela F, McIntosh RD (2021) Polymetallic Group 4 Complexes: Catalysts for the Ring Opening Polymerisation of rac-Lactide. Catalysts 11:551. https://doi.org/10.3390/catal11050551

3. Pedersen DS, Rosenbohm C (2001) Dry column vacuum chromatography. Synthesis (Stuttg) 2431–2434. https://doi.org/10.1055/S-2001-18722

4. Taylor D, Malcomson T, Zhakeyev A, Cheng S, Rosair GM, Marques-Hueso J, Xu Z, Paterson MJ, Dalgarno SJ, Vilela F (2022) 4,7-Diarylbenzo[c][1,2,5]thiadiazoles as fluorophores and visible light organophotocatalysts. Organic Chemistry Frontiers 9:5473–5484. https://doi.org/10.1039/D2QO01316A

5. Bratton D, Brown M, Howdle SM (2005) Tin(II) Ethyl Hexanoate Catalyzed Precipitation Polymerization of ε-Caprolactone in Supercritical Carbon Dioxide. Macromolecules 38:1190–1195. https://doi.org/10.1021/ma0484072
